# Immunological significance of survival-related alternative splicing in uveal melanoma

**DOI:** 10.18632/aging.203842

**Published:** 2022-01-19

**Authors:** Deqian Kong, Li Li, Huajun Wang, Ke Li, Guangying Zheng

**Affiliations:** 1 Department of Ophthalmology, The First Affiliated Hospital of Zhengzhou University, Zhengzhou 450052, Henan, China

**Keywords:** alternative splicing, immune infiltration, immune checkpoint blockade genes, uveal melanoma

## Abstract

Uveal melanoma (UM) is a highly malignant intraocular tumor. The imbalance of alternative splicing (AS) is a landmark of tumor initiation and progression. However, there are few studies of AS in UM. Thus, this study aimed to identify a new AS-based prognostic signature and reveal its relationship with tumor-infiltrating immune cells. Univariable Cox regression analysis identified survival-related AS events. The prognostic signature was constructed using the univariable and multivariable Cox regression analyses. Kaplan-Meier survival analysis, the proportional hazard model, and receiver operating characteristic curves verified its prognostic value. Single-sample gene set enrichment analysis was used to analyze immune cell enrichment. The correlation of the risk score with tumor-infiltrating immune cells and immune checkpoint blockade (ICB) genes was examined. We screened 2886 survival-related AS events, of which five were selected to build a prognostic predictor. The risk score was positively relevant with ICB key targets (HAVCR2, IDO1, and PDCD1) and the infiltration of T cells, MDSC, and activated B cells. We provided novel and effective indices, including a risk score and clinical nomogram, for prognostic prediction in UM and discussed the potential relationship between survival-related AS events and immune cell infiltration, which is crucial for developing immune-targeted therapy to improve prognosis.

## INTRODUCTION

Uveal melanoma (UM) is the most common primary intraocular malignant tumor in adults [1–3]. Most tumors infiltrate the choroid, and those localized to the iris and ciliary body are relatively seldom. A significant number of patients with UM will have systemic metastasis, with the most common metastatic organ being the liver; once metastasis occurs, survival is shortened, and surgical treatment becomes futile. When UM is large or invades its optic disc, surgery requires removal of the eye with radiotherapy, which causes great psychological suffering and loss of productivity among patients with UM [4], and this routine surgery cannot achieve satisfactory treatment effects. In contrast, tumor immunotherapy is considered a promising treatment method and is widely used in patients with cancer. However, no or limited response to immunotherapy is often observed in patients with UM [5–7]. Based on these considerations, this study aimed to identify appropriate targets for further treatment.

Alternative splicing (AS) events are very important post-transcriptional modifications and include alternate acceptor sites (AAs), alternate donor sites (ADs), alternate promoters (APs), alternate terminators (ATs), exon skips (ESs), retained introns (RIs), and mutually exclusive exons (MEs), which affect transcription and regulate many processes in the body [8, 9]. It exists in more than 90% of human genes and represents a significant factor in broadening the human proteome and increasing its diversity by producing different isomers, which can have different functions and lead to diverse diseases [10, 11]. Refer to previous research, AS events can apply critical process in tumor progression and can produce new epitopes for immunotherapy [12, 13]. Imbalances in AS can lead to a disordered microenvironment. On the one hand, AS events may lead to the production of specific mRNA transcripts in the tumor, thus activating cancer-related genes and pathways; on the other hand, it may also inhibit tumor immune escape, even killing cancer cells [14]. Therefore, the abnormal splicing products of cancer cells in the human body are potential new immunogenic targets [15]. Studies have shown that AS events in cancer samples are more frequent than in normal samples, indicating that AS events have tremendous potential value in tumor therapy and can be used as biomarkers and therapeutic targets to provide new approaches to treatment.

UM is characterized by high malignancy and easy metastasis; thus, early identification of high-risk patients is crucial for improving both our biological understanding of UM and tumor prognosis. Recently, based on previous studies, alternative splicing can effect the process of UM [16]. AS events has been shown to be potentially relevant for immunotherapy, but whether it affects the immune system of patients with UM and whether it can be used as a target for diagnosis and treatment remain unknown. There are few studies on AS events in UM, so it is of great clinical significance to explore the potential relationships between AS events and immune cells in UM. Thus, this study aimed to identify a new AS-based prognostic signature and reveal its relationship with tumor-infiltrating immune cells in the UM microenvironment.

## RESULTS

### Screening of survival-related AS events

**Figure 1:**
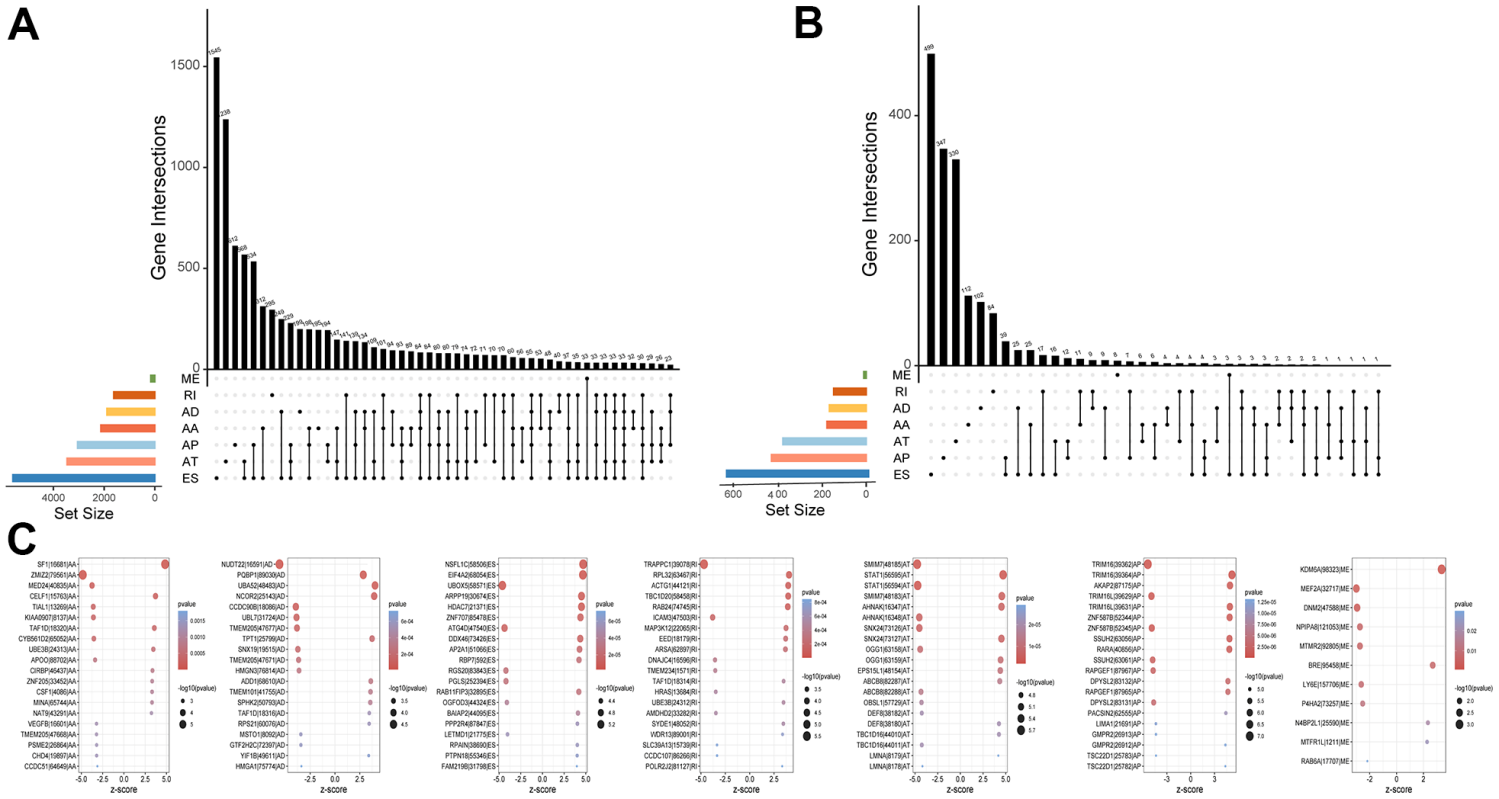
**Profiling of AS events in UM.** (**A**) The Upset plots of all AS events with UM. (**B**) The Upset plots of survival-relevant AS events. (**C**) The top 20 most significant survival-relevant AAs, ADs, ATs, APs, ESs, MEs and RIs is shown in the bubble chart.

**Figure 2:**
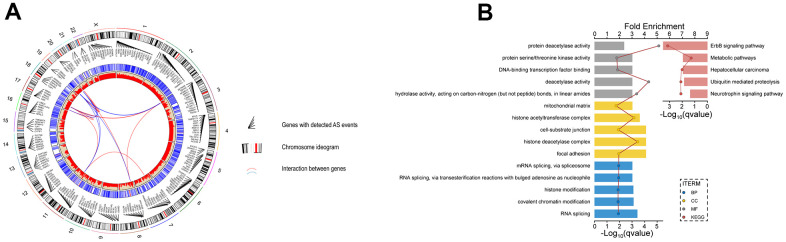
**Identification of AS events and the parent genes in UM.** (**A**) Circos plot show interaction relationship between survival-related AS events and their parent genes. (**B**) GO analysis show the enrichment of protein function, including biological process cellular component(BP), cellular component(CC) and molecular function(MF) and KEGG analysis show enrichment of pathways.

All AS events related to UM were screened (Figure 1A). A total of 2886 survival-related AS events were identified using univariable Cox regression analysis (Figure 1B and Supplementary Table 1). The splicing subtypes for all events and survival-related events are depicted in the upset plot. Among survival-related AS events, ES, AP AD, and AA occurred more frequently, ES being the most prevalent and ME being the least common splicing type. The top 20 survival-related AS events are shown in Figure 1C. The size of the dots represents the log10 of the P-value, and the color of the dots represents the P-value. The Circos plot shows the relationship between AS and the corresponding genes in UM (Figure 2A). Gene Ontology (GO) analysis of the genes involved showed enrichment of mRNA processing, cell-substrate adhesion, metabolic processes, and protein processing. The significantly enriched Kyoto Encyclopedia of Genes and Genome (KEGG) pathways included the ErbB signaling pathway, metabolic pathways, and hepatocellular carcinoma, which play important roles in the initiation and progression of many types of malignant tumors (Figure 2B). In summary, the above results show that AS and its corresponding genes play an important role in the biological processes relevant to UM.

### Identification of optimal survival-related AS events

**Figure 3:**
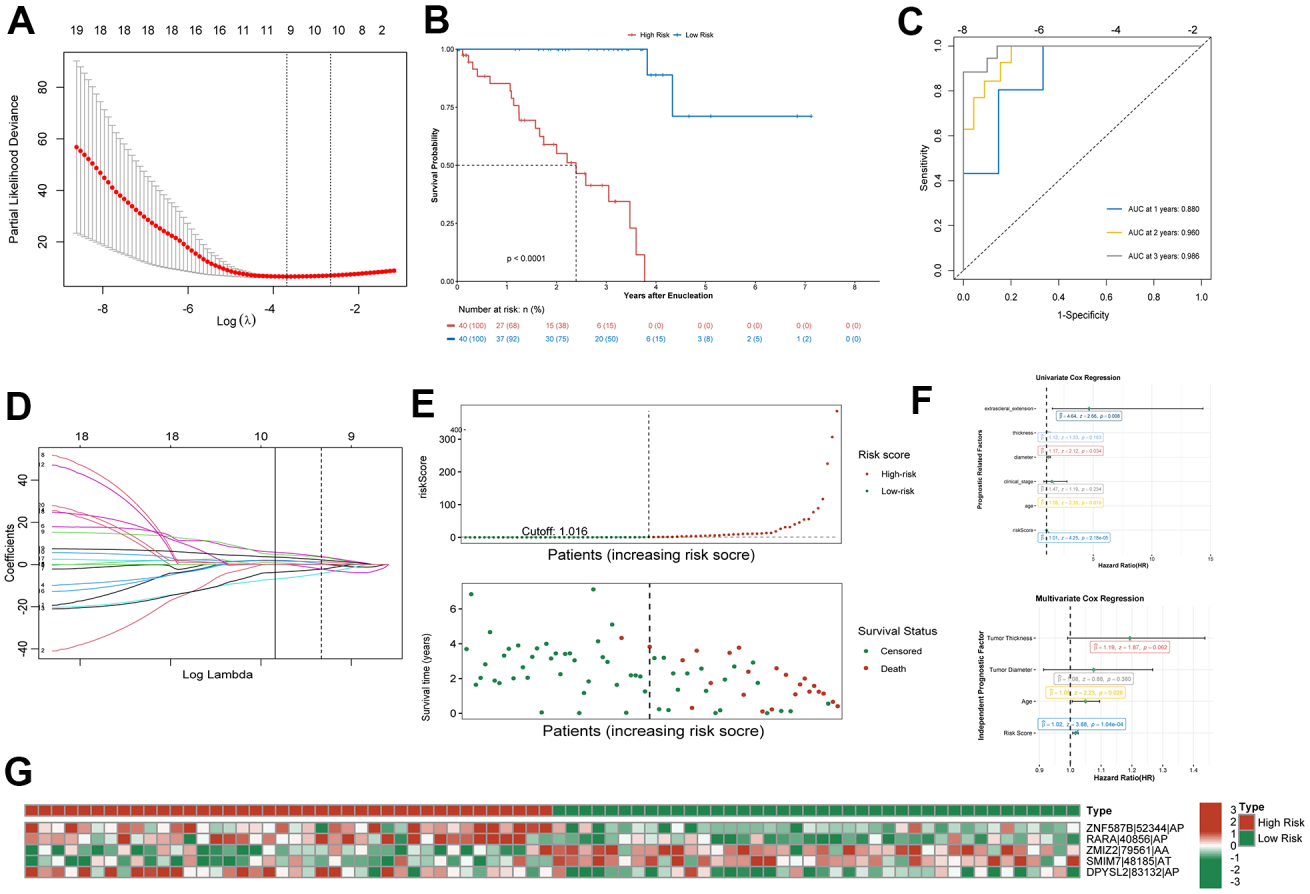
**Confirmation of prognostic signature.** (**A**) Parameter selection in the lasso regression. (**B**) Kaplan–Meier curve show survival in the high-risk and low-risk groups. (**C**) ROC analysis predict risk score for 1-, 2-, and 3-year overall survival. The AUC was calculated for ROC curves. (**D**) LASSO coefficient profiles of survival-related AS events. (**E**) Distribution of risk score and the survival time of UM patients. (**F**) Proportional hazards model results show Univariate Cox regression and Multivariate Cox regression results. (**G**) Heatmap of the survival-related AS events PSI value in UM.

Least absolute shrinkage and selection operator (lasso) regression was used to reduce the number of AS events related to prognosis, obtaining a model with the lowest lambda error and the highest accuracy (Figure 3A, 3D). The AS events thus selected were as follows (see Methods for the notation): ZNF587B|52344|AP, SSUH2|63056|AP, RARA|40856|AP, SF1|16681|AA, ZMIZ2|79561|AA, SMIM7|48185|AT, NSFL1C|58506|ES, TRAPPC1|39078|RI, and DPYSL2|83132|AP|. These nine AS events were included in the multivariable Cox regression analysis, and ultimately five AS events, shown in Table 1, were used to calculate the risk score, as detailed in the Methods. As shown in the heatmap of trend of change and calculation method passed percent spliced in (PSI) value (Figure 3G), ZNF587B|52344|AP, RARA|40856|AP, and DPYSL2|83132|AP are AS events associated with high risk, while SMIM7|48185|AT and ZMIZ2|79561|AA are associated with low risk.

**Table 1:** Prognostic signature of UM.

**ID**	**Z**	**HR**	**HR.95L**	**HR.95H**	**pvalue**
ZNF587B|52344|AP	4.894414405	1840.036562	90.66288692	37344.2173083333	9.85989E-07
RARA|40856|AP	4.856275588	1047298.986	3894.08933	281666667.700812	1.19614E-06
ZMIZ2|79561|AA	-4.829716885	7.15503E-05	1.48716E-06	0.00344243611386265	1.36727E-06
SMIM7|48185|AT	-4.761704539	0.000227908	7.22066E-06	0.00719351066995222	1.91965E-06
DPYSL2|83132|AP	4.678105765	163.5174741	19.32669503	1383.47318539684	2.89537E-06

### Establishment of a prognostic signature

Using the median risk score as the cutoff value, the patients were divided into a high-risk group (n = 40) and a low-risk group (n = 40) for further study (Figure 3E). Kaplan-Meier survival curve showed that the overall survival (OS) prognosis of the low-risk group was significantly better than that of the high-risk group (Figure 3B). The receiver operating characteristic (ROC) curve analysis showed that the area under the curve (AUC) for 1- 2-, and 3-year OS exceeded 0.75 (Figure 3C). This indicated that the prognostic model could accurately predict the survival status of patients. Univariable and multivariable Cox regression analysis showed that age and risk score could be used as independent prognostic indicators of OS in UM, among which the risk score had the best predictive ability (Figure 3F). Although the predictive value of other indices was limited, their potential clinical value should not be ignored.

### Construction of a clinical prognostic nomogram

**Figure 4:**
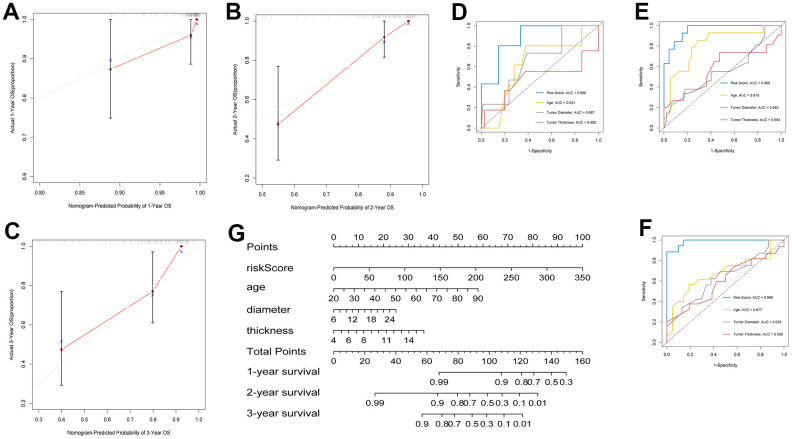
**Construction of clinical prognostic nomogram.** (**A**–**C**) 1-, 2-, and 3-year nomogram calibration curves. (**D**–**F**) AUC for predicting 1-, 2-, and 3-year survival with different clinical characteristics and risk score. (**G**) Nomogram was assembled by clinical characteristics and risk score for predicting 1-, 2-, and 3-year survival of UM patients.

A prognostic map, including risk score and clinical variables, was established to predict the prognosis of patients with UM. Among various clinical features, age, tumor diameter, tumor thickness, clinical stage, and extrascleral extension were used as candidate prognostic factors to explore whether they were the best prognostic indicators, and the AUC curves of 1-, 2-, and 3-year OS were analyzed as prognostic indicators. The AUC values of the risk score all exceeded 0.75 (Figure 4D–4F). The results showed that risk score was the best independent prognostic indicator. In nomogram, the score of each independent predictor in the line chart is the score of the corresponding upper score scale (points), and the total score of each subject (total points) is the sum of the scores of each independent predictor. The value of the total score on the risk axis of UM occurrence is the survival time for UM. The higher the total score, the lower the corresponding 1-year, 2-year and 3-year OS (Figure 4G). The line chart shows that the calibration curve is approximately diagonal, indicating strong stability in predicting prognosis (Figure 4A–4C).

### Correlation between tumor-infiltrating immune cells and risk score in UM based on ssGSEA

**Figure 5:**
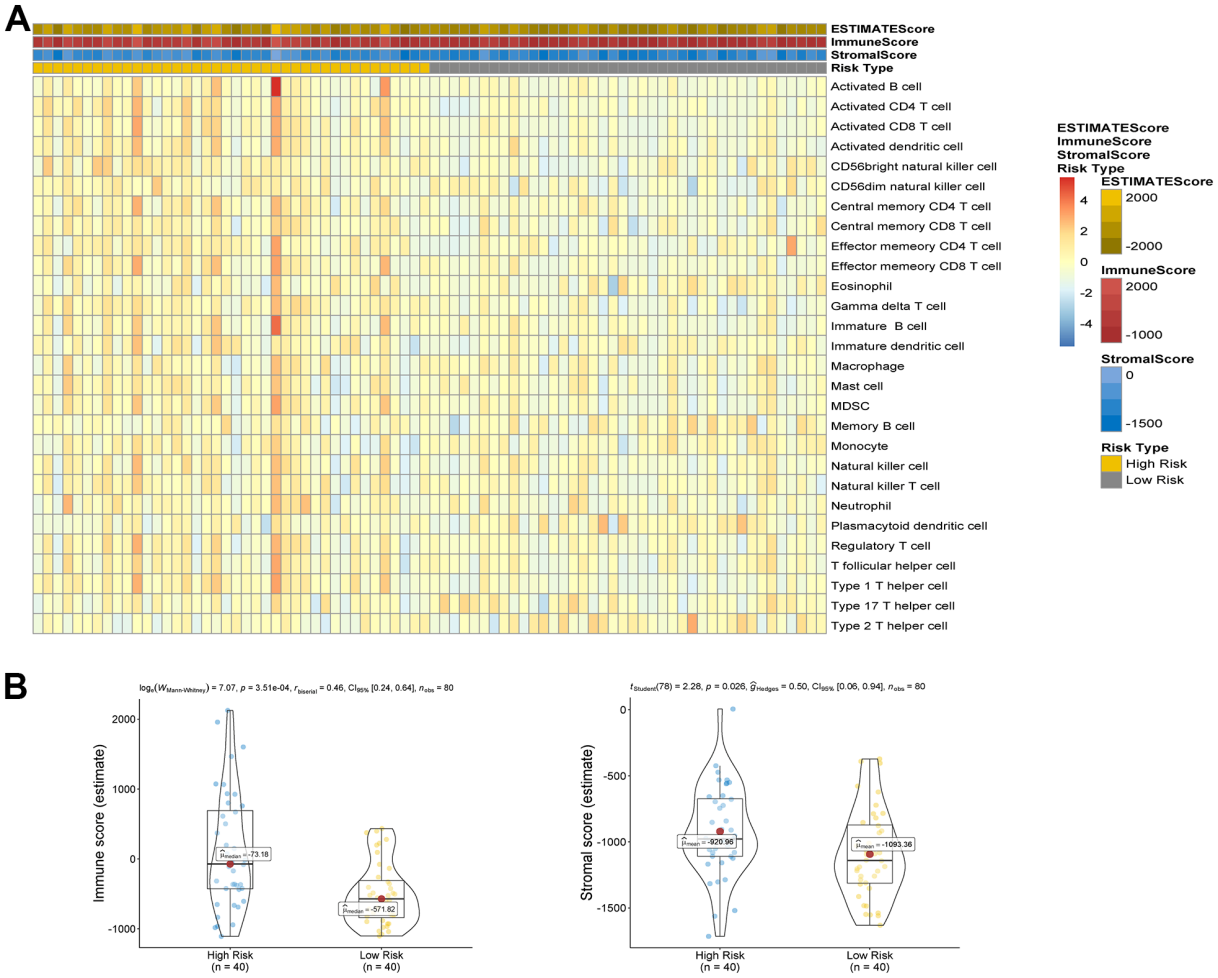
(**A**) Comparison of ESTIMATE score, stromal score, immune score and 28 types of immune cells ssGSEA enrichment between high/low-risk groups. (**B**) Comparison of stromal score and immune score between high/low-risk groups.

**Figure 6:**
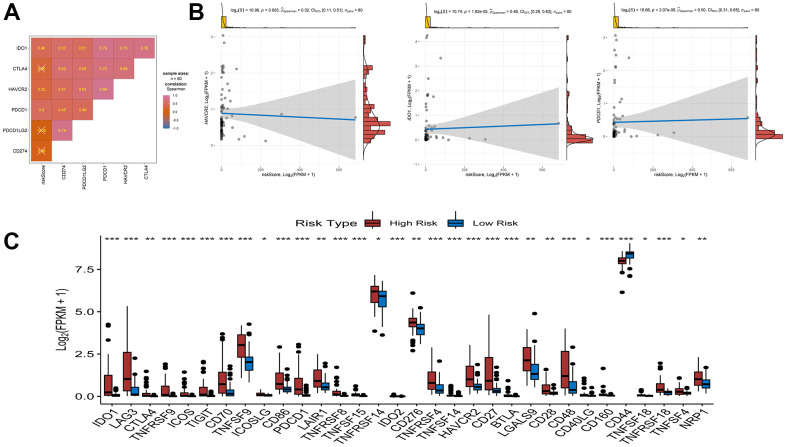
**Association between riskscore and immune check point genes.** (**A**) association analyses between six immune check point genes and risk score. (**B**) association between risk score and HAVCR2, association between risk score and IDO1, association between risk score and PDCD1. (**C**) Comparison of immune checkpoint blockade-related genes expression levels between high/low-risk groups.

**Figure 7:**
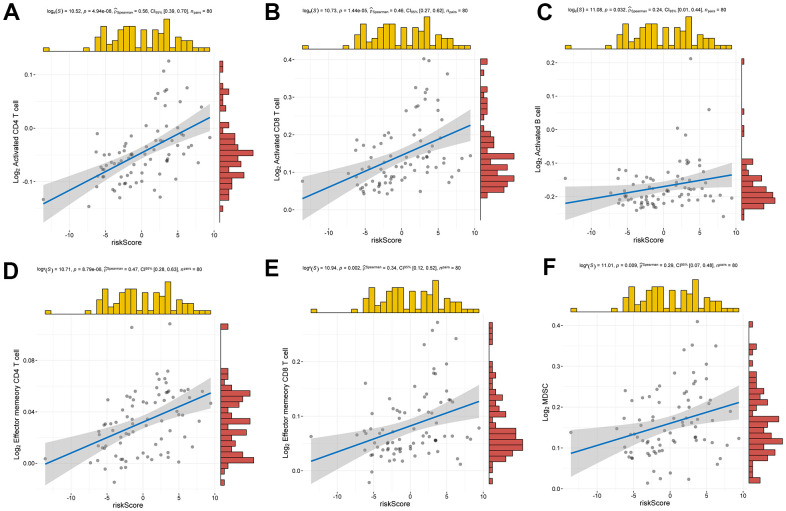
**Relationship between risk score and infiltrating immune cells.** (**A**–**F**) Positive correlation between risk score and activated CD4 T cell, activated CD8 T cell, activated B cell, effector memory CD4 T cell, effector memory CD8 T cell, MDSC.

The analysis of ESTIMATE immune and stromal scores showed that the high-risk and low-risk groups had significantly different characteristics in terms of the tumor immune microenvironment (Figure 5A). The corresponding heatmap showed that the high-risk group had higher immune scores and contained more immune cells than the low-risk group. Similarly, low-risk patients had higher stromal scores, which means less immune infiltration, and there was a significant difference in immunity between the two groups. We analyzed the two groups. The immune score and risk score were statistically significant in the high and low risk groups (Figure 5B). Adopting single-sample gene set enrichment analysis (ssGSEA) [15] analyzing the correlation between 28 types of immune cells and the risk score, we found that the risk score was positive correlated with six types of immune infiltrating cells, including activated CD4 T cells, effector memory CD4 T cells, central memory CD4 T cells, activated CD8 T cells, effector memory CD8 T cells, central memory CD8 T cells, myeloid-derived suppressor cells (MDSCs), and B cells (Figure 6A–6F). Moreover, the parent genes corresponding to the five survival-related AS events contributing to the risk score were also significantly associated with tumor-infiltrating immune cells (Figure 7), which provides a new way to clarify the characteristics of the UM immune regulatory network.

### Correlation between risk score and key molecules of ICB therapy

**Figure 8:**
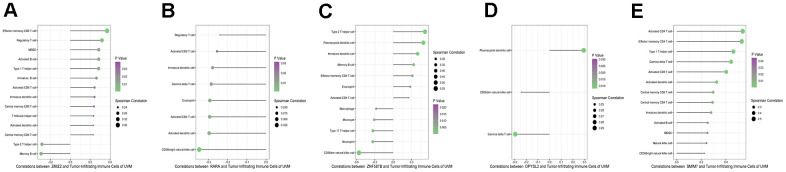
Correlation infiltrating immune cells with five parent genes (**A**–**E** stands for ZNF587B, RARA, DPYSL2, SMIM7, and ZMIZ2).

Immune checkpoint blockade (ICB) therapy is an effective treatment that has changed clinical decisions in oncology to a great extent. We associated six key immune checkpoint inhibitor genes (PDCD1, CD274, PDCD1LG2, CTLA-4, HAVCR2, and IDO1) with risk scores to reveal the potential risk markers of ICB in the treatment of UM (Figure 8A, 8B). The results showed significant positive correlations of the risk score with HAVCR2, IDO1, and PDCD1, suggesting that the risk score may play an important role in immunotherapy. Further analysis showed that 31 of the 47 immune blocking-related genes (including PD-L1) were significantly upregulated in high-risk patients (Figure 8C).

### Regulation network of SFs and AS

**Figure 9:**
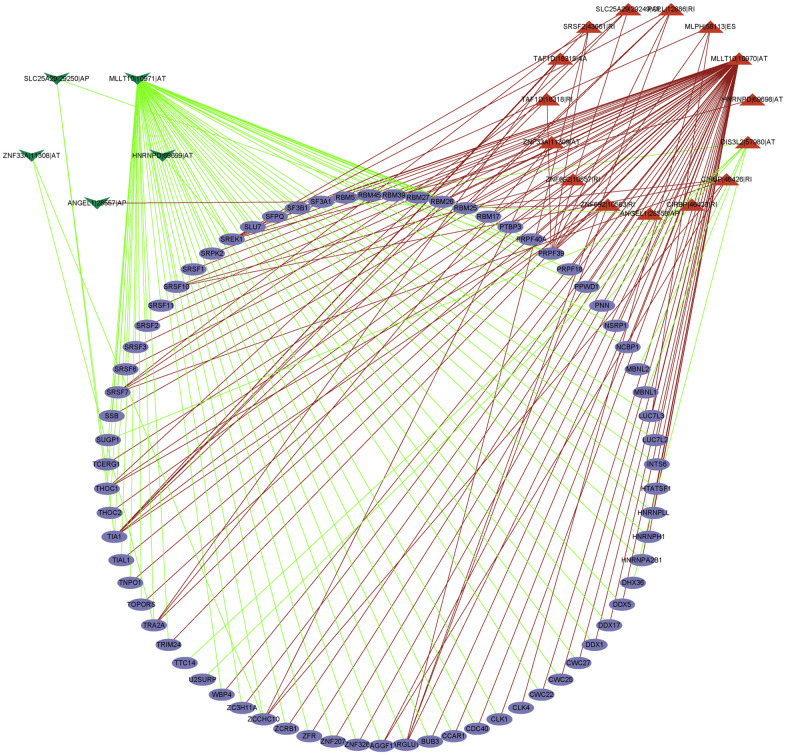
**The regulatory network between SFs and survival related AS events.** The positive/negative correlations between SFs and AS events by red/green line.

The interaction network between AS prognosis and splicing factors (SFs) was constructed based on correlation analysis to determine the potential mechanisms underlying AS alterations (Figure 9). There were five negative correlation with AS events (green arrows), 15 positive correlation with AS events (red arrows), and 71 SFs (purple ellipses) involved in the network. Among significant correlations, the strongest positive one was between TRA2A and MLLT10|10970|AT and the strongest negative one between TTC14 and DIS3L2|57980|AT. These results suggest that SFs are key regulatory factors participating in the regulation of AS events to further mediate the initiation and progression of UM.

## DISCUSSION

Currently, multiple lines of evidence, such as genomic complexity and epigenetic diversity among tumors, show UM to be a highly heterogeneous malignant with the characteristics of low survival rate and life-threatening tumor from a molecular and clinical standpoint [17, 18], a fact that needs to be taken into account by ophthalmologists. As a key post-transcriptional modification, AS events can regulate and modify RNA and can enlarge genomic coding [19]. Abnormal AS events can lead to cancer. In this study, survival-related AS events were screened using univariable and multivariable Cox regression analyses, and a prognostic signature for patients with UM was developed. Kaplan-Meier survival analysis, Cox regression analysis, and ROC curve analysis strongly proved the effectiveness of the proposed AS events prognostic signature. In addition, we constructed a clinical prognostic model using risk scores and established a clinical nomogram model composed of age, tumor diameter, thickness, and risk score. This line chart has great potential in clinical application and can accurately predict the survival rate of UM patients. The selected SFs are promising potential factors involved in the imbalance of AS events in UM and the establishment of a tumor-promoting/inhibiting microenvironment.

We expect to alleviate or improve the prognosis of patients with UM through immunotherapy, so there is an urgent need to develop powerful prognostic tools to predict its outcome. Abnormal AS events can cause abnormal infiltration of immune cells. In this study, a high density of immune cell infiltration, active immune state, and low survival rate were found in the high-risk group. We associated the risk score with 28 types of immune cells and found it is positively correlated with the infiltration of T cells (including activated/memory T cells), MDSCs, and activated B cells. In addition, studies have shown that tumor-infiltrating T cells can kill tumor cells and play an active role in the antitumor immune response. The evidence indicates that the growth of T-cell density is a favorable indicator of prognosis in non-small cell lung cancer, ovarian cancer, and glioblastoma [20]. However, unlike in other tumors, the growth of T-cell abundance in UM revealed the opposite effect [21]. Based on the results of our study, abnormal AS events may mediate undiscovered T-cell subsets or lead to tumor-infiltrating T-cell dysfunction, which can be a key target for immunotherapy [22]. We speculate that AS events may drive site-specific antigen heterogeneity associated with T-cell infiltration, resulting in sustained T-cell exposure damage or regulatory T-cell production. In addition, there was a positive correlation between MDSCs and the risk score. Malignant tumors are often in a state of immunosuppression. MDSCs are very typical immunosuppressive cells and it can inhibit the activity of natural killer cells (NK cells) and both adaptive and innate immune response [23]. NK cells are effector cells with superior natural immune function and the main not directly T cell dependent antitumor immune cells, and the destruction of the function of NK cells induces liver metastasis of UM [24, 25]. Enrichment of these immune cells indicates poor prognosis. Infiltrating B cells play crucial role in promoting tumor immunity. However, not all B cells can have a positive immune response in tumor [26]. Regulatory B cells produce anti-inflammatory cytokines such as interleukin (IL)-10 to negatively regulate the immune response and play an anti-tumor role [27]. There are few studies on infiltrating B cells in UM, which needs to be further developed; therefore, these immune cells maybe be used as a potential and promising immune target.

Radiotherapy can expand the number of T cells and recruit T cells to the irradiated site, so that the irradiated cells are more vulnerable to T cell-mediated cell killing. Combined with immunotherapy, it can enhance the anti-cancer effect [28]. At present, this treatment shows great result in patients with melanoma [29], and radiotherapy combined with immunotherapy may be more appropriate for patients with uveal melanoma with poor metastatic immune effect.

Genes with splicing events can activate immune cells [30] and may be used as gene signatures for various cancer prognostic biomarkers [31, 32]. Our study revealed that the five key signature genes (ZNF587B, RARA, DPYSL2, SMIM7, and ZMIZ2) corresponding to abnormal splicing events are related to immune cells. ZNF587B is an important member of the C2H2-type zinc finger protein (ZFP) family. ZNF587B is also an important transcription factor that has been found to be related to ovarian cancer and may be a therapeutic target [33]. The PML-RARA fusion gene is related to acute ovarian cancer, and RARA participates in estrogen signal transmission and is the target gene of estrogen in breast cancer [34]. DPYSL2 is related to the mTOR signaling pathway; mTOR, as a central regulator of proliferation signal transduction, is an ideal target for tumor treatment [35]. SMIM7 inhibits apoptosis in liver cancer cells [36]. ZMIZ2 is a PIAS-like protein involved in prostate and colorectal cancer where it promotes tumor growth [37]. These parent genes are important transcription factors or are related to tumor treatment targets, but considering individual genes may lead to biased results in the analysis of correlation with immune cells. The risk score is obviously a better indicator of correlation with immune cells, but all prognostic markers provide a new possibility for elucidating the immune network in UM. This is worth noting in the process of searching for new antigens of tumor mutations. These genes are related to infiltrating immune cells, including both tumor promoting and tumor inhibiting immune cells. AS events and their parent genes may become new epitopes, which can expand the target of immunotherapy for malignant tumors and help formulate targeted immunotherapy strategies, which is the focus of our further analysis in the future.

Furthermore, it is well known that UM and skin cutaneous melanoma (CM) have similar cell sources, and currently, promising immunotherapies, such as ipilimumab (anti-CTLA4), nivolumab (anti-PD1), and durvalumab (anti-PDL1), are being successfully applied for the clinical treatment of CM, with great improvements in patient survival rates [38, 39]. However, these drugs have no positive effect on the treatment of UM either alone or in combination. UM has a unique immunological profile. Our results showed that the risk score was significantly and positively correlated with the expression levels of 31 ICB-related genes and 3 ICB key targets and that TIM3 and LAG3 interfere with antitumor immunity in UM; these two genes are also highly expressed in our high-risk group. It is believed that an increasing number of immune genes will be discovered in the future, and inhibitors of immune checkpoint genes are expected to block the immune escape of tumor cells, thus allowing the immune system to kill cancer cells. In summary, it is suggested that UM is suitable for targeted immunotherapy strategies. Based on the risk score, prognostic markers play an important role in improving the prognosis of patients with UM.

To explore the upstream regulatory mechanism of AS events, we constructed an interaction network between survival-related SFs and prognostic AS events. Our exploration of upstream and downstream regulation mechanisms will contribute to explaining how AS plays a role in UM. In particular, we determined that AS events were closely related to ICB genes and immune cell infiltration. At present, the effect of UM in ICB treatment is not satisfactory. However, there is still great potential space for immunotherapy. We have explored valuable variable shear events and screened prognostic signature. The immune cells stimulated and induced by them and how to affect UM and finally eliminate tumors will be the focus of our future research work. This preliminary work is very important as it enables us to explore the follow-up immunotherapy of UM, developing targeted immunotherapy, and improving prognosis.

In conclusion, exploring the effect of AS events on the prognosis of patients with UM, we constructed effective prognostic markers and clinical predictive models, which suggest the clinical prognostic value of AS events. We systematically analyzed the complete upstream and downstream regulatory mechanisms of AS and immune cell-related AS events. Finally, we established a survival-related AS-SFs regulatory network for exploring the potential mechanism involved in UM, providing new clues for studying the pathogenesis of UM and improving its prognosis.

## MATERIALS AND METHODS

### Data acquisition and processing

Clinical and transcriptome information of 80 UM cases were downloaded from The Cancer Genome Atlas (TCGA) database (http://cancergenome.nih.gov). The TCGAspliceseq database (http://bioinformatics.mdanderson.org/TCGASpliceSeq) was used to download AS data related to UM and specifically the PSI values representing the occurrence probability of different AS events (from 0 to 1). The PSI can quantify AS events in a way suitable for further analysis. In this study, we selected the AS events with PSI exceeding 0.75.

### Normalization and annotation of AS events

For the comprehensive and unified comparison of different AS events, a formalized annotation was devised, consisting of the parent gene involved, the ID number of the AS event, and the splicing type (e.g., “SF1|16681|AA”). AS events include the seven different AS modes (AA, AD, AP, AT, ES, RI, and ME).

### Selection of survival-related AS events

Univariable Cox regression analysis was used to screen out AS events related to the OS of patients with UM, excluding the AS events with standard deviation of the PSI value less than 0.01. Considering that one gene may correspond to multiple splicing modes, an upset plot was created to visualize the interaction set of AS events, and a bubble chart was used to visually display the top 20 survival-related AS events. Enrichment analysis of the associated genes using GO and KEGG was performed to analyze the enrichment of genes involved in terms of protein function(biological process cellular component(BP), cellular component(CC) and molecular function(MF)) and pathways, respectively. In addition. In addition, Circos plot visualization was used to show the correlation between survival-related events and the corresponding genes more directly.

### Construction and verification of a prognostic signature

Lasso regression analysis was used to improve the accuracy of the model while preventing model overfitting. The identified AS events were included in the multivariable Cox regression analysis to analyze the prognostic signature. All selected AS events were fitted to calculate the risk score. The formula is as follows:

risk score = β_ASevent1_ × PSI_ASevent1_+β_ASevent2_×PSI_ASevent2_+…+β_ASeventn_×PSI_ASeventn_.

Patients were divided into a high-risk and a low-risk group based on a risk score cutoff, and the survival of the two groups was analyzed using Kaplan-Meier survival curves. The ROC curve was used to examine the prognostic value of the signature. Cox regression was used to explore whether the risk score could be used as an independent factor for predicting prognosis.

### Establishment of a clinical prognostic nomogram

To predict the OS of patients with UM, we established an AS-pathologic nomogram including the risk score and clinical indices (age, tumor diameter, tumor thickness, clinical stage, and extrascleral extension), which was used to estimate the probability of 1-, 2-, and 3-year OS in patients with UM. A calibration curve reflecting the predictive value of the nomogram was plotted. To comprehensively evaluate the ability of risk score, age, tumor diameter, and tumor thickness to predict the prognosis of 1-, 2-, and 3-year OS, the AUC value was calculated using a time-dependent ROC curve to evaluate its accuracy [38].

### Correlation between prognostic signature and tumor-infiltrating immune cells

There are at least 28 kinds of infiltrating immune cells in the tumor. To reveal the infiltration of immune and stromal cells in UM, single-sample gene set enrichment analysis(ssGSEA) was used to analyze the enrichment of 28 kinds of immune-related cells in the high- and low-risk groups [39], and the stromal cells and immune cells were compared in the two risk groups. The correlation between risk score and 28 types of immune-related cells was analyzed, as well as the correlation between parent genes corresponding to five AS events and tumor-infiltrating immune cells.

### Risk score and ICB treatment

ICB treatment involves six key genes, namely programmed death 1 (PD-1, also called PDCD1), programmed death ligand 1 (PD-L1, also called CD274), programmed death ligand 2 (PD-L2, also called PDCD1LG2), cytotoxic T-lymphocyte antigen 4 (CTLA-4), indoleamine 2,3-dioxygenase 1 (IDO1), and T-cell immunoglobulin domain and mucin domain-containing molecule-3 (TIM-3, also called HAVCR2) [40–42]. To explore the potential role of the risk signature in the ICB treatment of UM, we analyzed the correlation between the risk score and the expression of these six ICB key genes and compared the expression of 47 ICB-related genes between the high- and low-risk groups.

### Establishment of a correlation network between SFs and survival-related AS events shutdown

SFs as upstream factors could regulate a total of 404 events AS events [43], as shown in Supplementary Table 2. We analyzed the interaction between SFs and survival-related AS events. Using r > 0.82/ r < -0.82 as the cutoff values on correlation coefficients, and P < 0.001, Cytoscape software 3.8.2 was used to establish a regulatory network of the interaction between SFs and AS events with survival.

### Statistical analysis

In this study, R software was used for statistical analysis. The Wilcoxon test was employed to compare two groups, and the Kaplan-Meier log-rank test to analyze survival curves. Risk score, clinical indexes, density of tumor immune cell infiltration, ICB genes, SFs, and AS interaction were analyzed by Spearman correlation analysis. Cox regression, univariable and multivariable, and lasso regression were used to construct the prognostic model.

## Supplementary Material

Supplementary Table 1

Supplementary Table 2
