# Supplementary material for: Immunological significance of survival-related alternative splicing in uveal melanoma
Source: Aging (Albany NY). 2022 Jan 19;14(2):811–25. doi: 10.18632/aging.203842 (PMC8833124; doi:10.18632/aging.203842)
Supplement: Supplementary Table 1 [file aging-14-203842-s001.doc]

**Supplementary Table 1. A total of 2886 survival-related AS events.**

**ID**

TRIM16|39362|AP

TRIM16|39364|AP

AKAP2|87175|AP

TRIM16L|39629|AP

TRIM16L|39631|AP

ZNF587B|52344|AP

ZNF587B|52345|AP

SSUH2|63056|AP

RARA|40856|AP

SF1|16681|AA

ZMIZ2|79561|AA

SSUH2|63061|AP

SMIM7|48185|AT

RAPGEF1|87967|AP

STAT1|56595|AT

STAT1|56594|AT

NSFL1C|58506|ES

TRAPPC1|39078|RI

DPYSL2|83132|AP

RAPGEF1|87965|AP

EIF4A2|68054|ES

DPYSL2|83131|AP

UBOX5|58571|ES

SMIM7|48183|AT

AHNAK|16347|AT

AHNAK|16348|AT

SNX24|73126|AT

SNX24|73127|AT

ARPP19|30674|ES

HDAC7|21371|ES

OGG1|63158|AT

OGG1|63159|AT

PACSIN2|62555|AP

EPS15L1|48154|AT

ZNF707|85478|ES

ATG4D|47540|ES

LIMA1|21691|AP

GMPR2|26912|AP

GMPR2|26913|AP

DDX46|73426|ES

TSC22D1|25782|AP

TSC22D1|25783|AP

NUDT22|16591|AD

ABCB8|82287|AT

ABCB8|82288|AT

OBSL1|57729|AT

AP2A1|51066|ES

CLASP2|63869|AP

DEF8|38182|AT

DEF8|38180|AT

TBC1D16|44010|AT

TBC1D16|44011|AT

NDUFC1|70617|AP

NDUFC1|70618|AP

ZNF677|51699|AP

ZNF677|51698|AP

RBP7|592|ES

LIMA1|21688|AP

RGS20|83843|ES

PGLS|252394|ES

RAB11FIP3|32895|ES

LMNA|8179|AT

LMNA|8178|AT

CHCHD3|81833|AT

CHCHD3|81834|AT

GALK2|30516|AP

THAP4|58391|AP

THAP4|58392|AP

OGFOD3|44324|ES

PQBP1|89030|AD

BAIAP2|44095|ES

OBSL1|57728|AT

CLN3|35707|AP

CLN3|35708|AP

PI4K2A|12728|AP

PI4K2A|12729|AP

PER1|39092|AP

MAP3K12|22063|AP

MAP3K12|22061|AP

PISD|61880|AP

RPL32|63467|RI

UBA52|48483|AD

PPP2R4|87847|ES

PISD|61881|AP

LETMD1|21775|ES

RPAIN|38690|ES

RFX7|30752|AT

RFX7|30750|AT

PDXK|60786|AP

RPL31|54729|AT

PDXK|60785|AP

ITGB3|42069|AT

PTPN18|55346|ES

RPL31|54732|AT

RAP1GAP|981|AP

PLEKHB1|17699|AP

ZNF540|49542|AP

ASTN2|87366|AP

MITF|65587|AP

FAM219B|31798|ES

RAP1GAP|980|AP

ACTG1|44121|RI

TBC1D20|58458|RI

GALK2|30518|AP

NCOR2|25143|AD

BCAS3|42872|ES

TBC1D8B|89808|AT

CCDC90B|18086|AD

SSBP4|48426|AP

SSBP4|48427|AP

ZNF568|49430|AP

UBXN11|1254|ES

RAB24|74745|RI

NISCH|65221|AT

NISCH|65220|AT

PHF19|87401|AT

PHF19|87402|AT

UBL7|31724|AD

NRG2|73626|AP

ACOT9|88694|ES

DIDO1|60091|AT

LETMD1|21753|ES

ORAI3|36207|AT

ORAI3|36208|AT

DMKN|49138|AT

MADD|15722|ES

EPC1|11156|AP

SERPING1|15865|AP

SERPING1|15866|AP

TMEM205|47677|AD

EPC1|11157|AP

SNAPC5|31269|AT

SNAPC5|31270|AT

DMKN|49134|AT

TBC1D8B|89806|AT

APP|60283|ES

SF1|16682|ES

MGAT5B|43693|AP

MGAT5B|43694|AP

ICAM3|47503|RI

C2CD5|20734|ES

NCK1|66940|AP

PTPN18|55344|ES

FAM49B|85138|AP

ZNF540|49541|AP

GSTO2|13049|AP

SLC38A6|27793|ES

LETMD1|21759|ES

AUH|86823|AT

AUH|86822|AT

PPP4R1|44611|ES

NT5C3A|79217|ES

NRG2|73625|AP

DIDO1|60092|AT

IGSF3|4366|ES

EEF1G|16351|AP

EEF1G|16350|AP

NEDD9|75340|AT

NEDD9|75339|AT

AES|46658|AP

MLPH|58112|AP

MLPH|58111|AP

MED24|40835|AA

TMX2|15920|ES

TPT1|25799|AD

SNX19|19515|AD

MEIS3|50642|AT

MEIS3|50643|AT

MAP3K12|22065|RI

CCND3|76156|AP

PER1|39090|AP

PPP1R7|58332|AT

PPP1R7|58333|AT

MAP3K7|77020|ES

PCBP2|22045|AP

PCBP2|22043|AP

CELF1|15763|AA

SYNE2|27846|ES

TMEM8B|86317|AP

CDH23|12059|AT

MAP2K5|31333|ES

EED|18179|RI

RPS24|12295|ES

TUBB4A|47062|ES

CD46|9662|ES

ZNF519|44756|AT

PDE4D|72138|AP

CD47|66014|ES

CCND3|76154|AP

SLC7A8|26710|AP

FAM49B|85137|AP

ARSA|62897|RI

LRRC29|36979|AP

TMEM205|47671|AD

C11orf1|18686|AP

LGALS8|10382|ES

ZNF568|49429|AP

MFF|57813|ES

WIPI2|78656|ES

NFIC|46676|AP

PPM1B|53413|AT

DAP3|8116|AP

SEC23A|27347|AT

SLC7A8|26712|AP

LETMD1|21751|ES

CRTC1|95073|ES

HECTD2|12517|AT

HECTD2|12516|AT

USP48|996|AT

USP48|997|AT

LY6E|85381|ES

MYO1G|79577|AT

MYO1G|79578|AT

NFIC|46675|AP

GSTO2|13047|AP

HMGN3|76814|AD

ARSE|88421|AP

ASTN2|87367|AP

NCK1|66941|AP

FAM120C|89237|AT

FAM120C|89238|AT

ATG4D|47541|ES

ARSE|88420|AP

ASPM|9286|ES

ADD1|68610|AD

TAOK2|35998|AT

TAOK2|35999|AT

GIGYF2|58026|ES

ATG4B|58402|ES

ENTPD1|12651|AP

TIAL1|13269|AA

SHMT2|22539|AP

SEC23A|27348|AT

TM4SF19|68240|AT

UBXN11|1256|ES

DCAF8|8436|AP

DCAF8|8438|AP

DAP3|8117|AP

HSPA14|10830|AT

HSPA14|10831|AT

KIAA0907|8137|AA

TMEM205|47674|ES

TMEM101|41755|AD

CHCHD6|122683|ES

IDS|90294|ES

TIRAP|19384|AT

PHF20L1|85192|AT

PHF20L1|85193|AT

FAM86B1|82693|ES

RNF135|40140|ES

SHC1|7855|AP

SHC1|7854|AP

CTBP2|13410|AP

ZNF33A|11308|AT

ZNF33A|11309|AT

TAF1D|18320|AA

MANBAL|59343|ES

CEP63|66877|AT

CEP63|66878|AT

SPHK2|50793|AD

FHL2|54835|ES

PPM1B|53414|AT

ABR|38287|AP

MTA3|53364|AP

CPEB2|68798|ES

HNRNPLL|53259|AT

DNAJC4|16596|RI

TMEM150A|54299|AP

TJP1|29766|ES

TANK|55730|AP

EXD3|88297|AT

ING4|19914|ES

TXLNA|1558|AP

CYB561D2|65052|AA

TMEM234|1571|RI

ASCC2|61680|ES

C11orf1|18688|AP

DMPK|50523|ES

ARMCX2|89664|AP

PAK1|17951|AP

ARMCX2|89665|AP

NPIPA5|34150|ES

FLT3LG|50941|AP

SPATA20|42435|ES

GART|60431|AT

GART|60430|AT

FAM86B1|82685|ES

HOGA1|12727|ES

B9D1|39710|AT

SP100|57896|AT

ZNF451|76583|AT

ABCC6|34220|AT

ABCC6|34219|AT

LETMD1|21754|ES

STAG2|90037|ES

RNF135|40138|ES

EFCAB2|10480|AT

EFCAB2|10479|AT

ZNF451|76581|AT

EVL|29241|AP

NEDD4L|45651|AP

COA1|79332|AT

CLSTN1|575|ES

SEC14L1|43703|AP

SYNRG|40526|ES

GEMIN7|50398|ES

PPAN|47461|AT

PPAN|47460|AT

TAF1D|18314|RI

SPATA20|42431|ES

FBLN1|62666|AT

ZFP64|59813|AT

FAM86B1|82707|ES

HRAS|13684|RI

UBE3B|24312|RI

LYRM4|75241|AT

LYRM4|75240|AT

AMDHD2|33282|RI

ANKRD42|18051|AT

TAF1D|18316|AD

KIAA0391|27213|AP

KIAA0391|27214|AP

CIRBP|46438|ES

SYDE1|48052|RI

TANK|55731|AP

BCAP29|81357|AT

PAK1|17950|AP

MPRIP|39457|ES

UBE3B|24313|AA

CEPT1|4139|AP

ALAS1|65186|ES

RPS21|60076|AD

PVRL2|50348|AT

PVRL2|50349|AT

RUFY3|69445|AP

TNFSF13|38969|AP

TNFSF13|38970|AP

FBXO38|73980|ES

CEP63|66876|AP

MSTO1|8092|AD

MPPED2|14793|AT

MPPED2|14794|AT

GTF2H2C|72397|AD

SCLT1|70587|AT

SEPT4|42689|AP

INF2|29547|ES

AGTRAP|672|ES

TMEM91|50045|AP

MYO1B|56609|ES

HYAL1|64992|AP

SEPN1|1195|ES

SHMT2|22540|AP

SPATA20|42430|ES

YIF1B|49611|AD

DCTN6|83284|ES

TMEM8B|86318|AP

SHC4|30508|AP

LRRC8A|87787|AP

MAGI2|80214|AT

MAGI2|80215|AT

MTMR10|29793|ES

SARNP|22253|AT

APOO|88702|AA

NTAN1|100489|ES

MRPL33|53046|ES

PDXK|60789|AT

PDXK|60788|AT

SHC4|30507|AP

UBTF|41833|ES

HDLBP|58345|AP

HMGA1|75774|AD

MRPL45|40572|ES

WDR13|89001|RI

HYAL1|64993|AP

CRIP1|29652|AT

CRIP1|29653|AT

PLEKHB1|17697|AP

HHAT|9688|AP

SBNO2|46391|AP

WIZ|48090|ES

HYKK|32100|ES

DNASE1L1|90581|ES

RPS15A|34260|ES

SARNP|22251|AT

ERP29|24569|ES

KDM6A|98323|ME

BCAS3|42871|ES

CIRBP|46437|AA

AP4S1|27099|AT

AP4S1|27100|AT

FCGRT|50957|AP

FCGRT|50958|AP

GPR108|47073|AP

GPR108|47074|AP

SLC39A13|15739|RI

SCMH1|2053|ES

SSH2|40064|ES

CCDC107|86266|RI

LETMD1|21782|ES

C8orf59|84338|ES

TXLNA|1559|AP

POLR2J2|81127|RI

SDC1|52760|AP

CLASP2|63871|AP

MACF1|1881|ES

DYNLL1|24763|RI

MAPKAP1|87583|ES

ZNF185|90396|AP

OARD1|76091|AD

FMNL3|21605|ES

IMMP1L|14820|ES

EEF1E1|75282|AT

FNIP2|71000|AP

SAMD12|84992|AT

C9orf9|87993|AP

C9orf9|87992|AP

ZNF205|33452|AA

CAMTA1|507|ES

TSPAN4|13790|AP

NFIX|47901|AP

CCND3|76161|ES

PHF8|89235|RI

LRRFIP1|58131|AT

LRRFIP1|58130|AT

POLR2J3|81116|RI

SPIN2A|89298|AP

CSF1|4086|AA

CLPP|47023|AP

CLPP|47024|AP

TMEM150A|54297|AP

RTEL1|60146|AD

NCOR2|25144|ES

FAM189B|8050|ES

UBXN11|1257|ES

SAMD12|84993|AT

MINA|65744|AA

PDE7A|83988|AP

TIAL1|13268|ES

ABI1|11048|ES

ZDHHC11|71450|AT

LEPROTL1|83272|AT

LILRB4|51931|ES

DGUOK|54009|ES

FANCL|53654|AD

CITED1|89450|AP

PGF|28457|ES

PSMB5|26694|AD

SH3PXD2A|13021|AP

CAMK1|63175|ES

BCAP29|81359|AT

CTNNB1|64249|RI

MED31|38771|ES

TIRAP|19385|AT

CREM|11230|AP

RUFY3|69444|AP

PLEKHB2|55376|AD

SORBS1|12628|ES

ZDHHC7|37873|ES

CGREF1|52933|AT

CGREF1|52934|AT

NCOR2|25145|ES

FBLN1|62665|AT

SEC14L1|43701|AP

PKP4|55680|ES

LILRB4|51930|ES

C4orf19|69001|AT

LRRC29|36981|AP

C4orf19|69000|AT

SMIM19|83740|AD

CLEC16A|34006|AD

MRPL10|42104|ES

GAB1|70700|ES

CAMKK2|24854|ES

SEC31A|69728|ES

LETMD1|21763|ES

FDFT1|82650|RI

CAPN3|30155|ES

PLSCR1|67169|ES

NFATC1|46239|AT

NFATC1|46240|AT

ABI1|11041|ES

NDUFAF1|30087|ES

CARD16|18544|AP

CAPN3|99509|ES

RNF43|42672|AT

RNF43|42671|AT

YIPF1|3079|ES

SCLT1|70589|AT

NAT9|43291|AA

NSFL1C|58501|ES

BOLA3|54017|ES

AIG1|77970|AT

LIMK1|80059|AP

MACF1|1883|ES

IFI27L1|29056|AD

ASAP2|52611|ES

GOLIM4|67565|ES

PARD3|11211|ES

USP25|60221|ES

NTAN1|100490|ES

LRRC8A|87788|AP

TRIM37|42721|AT

VEGFB|16601|AA

TMEM205|47668|AA

DMTN|82928|ES

NECAB2|37802|AP

GAS8|38199|AP

NECAB2|37803|AP

PBRM1|65237|ES

ANKRD13A|24395|AP

TFDP1|26391|ES

SLC48A1|21358|AP

SCMH1|2047|ES

PSME2|26864|AA

SH3D21|1764|AP

CHD4|19897|AA

TRIM37|42723|AT

FBF1|43529|AT

FBF1|43530|AT

TP53I3|52811|RI

SLC25A26|65542|AT

SLC25A26|65543|AT

CAPN3|99508|ES

ROM1|16366|AP

ROM1|16367|AP

KAT7|42322|ES

PBRM1|65236|ES

AP2S1|50598|AP

ZFP64|59811|AT

ALG9|18682|AP

ALG9|18683|AP

APOC1|50356|AP

APOC1|50357|AP

SLC48A1|21359|AP

MAGED2|89247|AP

FGFR1OP|78436|ES

TMEM8B|86320|AT

TMEM8B|86321|AT

PDZD7|12862|AT

FAM126A|78946|ES

PLAUR|50230|AT

PLAUR|50231|AT

SLC25A45|16826|ES

LYRM9|39869|AD

RFNG|44268|AP

RFNG|44267|AP

VGLL4|63393|AP

WDR92|53826|AD

TMPO|23849|ES

SLC52A1|38647|AP

SLC52A1|38648|AP

LCORL|68866|AT

LCORL|68865|AT

ST7L|4207|AT

ST7L|4205|AT

SLMO1|44684|AP

PLEC|85513|AP

SMIM19|83739|AD

ACOT2|28301|AD

SH3D21|1763|AP

PTK2|85305|ES

AP2S1|50597|AP

FTSJ2|78605|ES

ELP3|83201|AP

ELP3|83202|AP

PLEC|85508|AP

ITGA7|22209|AP

STOML1|31624|ES

NEDD4L|45649|AP

UAP1|8751|ES

HAX1|7818|ES

GAS8|38200|AP

TCAIM|64356|AP

CNBP|66709|AD

TRAPPC9|85282|ES

SDC1|52759|AP

LETMD1|21761|ES

PTPRH|262450|ES

EXOC3|118858|ES

RREB1|75253|ES

PHF17|70578|AP

ZNF789|80648|AT

DCAF11|26830|AP

DCAF11|26831|AP

UGGT2|26129|AT

BRPF1|63155|ES

BSCL2|16400|AP

RPP38|10864|ES

FBXO18|10669|AP

ZNF789|80647|AT

AKIP1|14279|ES

ICMT|385|ES

CARD16|18543|AP

PLXNB2|62819|AD

UHRF1BP1L|23900|AT

UHRF1BP1L|23899|AT

PRRT4|81667|RI

CDK10|38119|ES

CCDC51|64649|AA

SLC27A1|48307|AP

SLC27A1|48308|AP

TCAIM|64355|AP

POLR2G|16420|RI

ANAPC5|24862|AA

S100A1|7741|AA

HEXIM2|100145|ES

MGAT5B|43699|ES

AFTPH|53773|ES

TRIM16|273917|ES

CNOT4|81885|AT

TMEM62|30214|ES

SGCD|74302|AT

SGCD|74304|AT

SUPT20H|25661|ES

PPP3CB|12156|AA

ZNF346|74699|AT

ZNF346|74698|AT

PDE7A|83989|AP

ETV4|41710|AP

SORBS1|12641|ES

TMEM242|78239|AT

TMEM242|78240|AT

TRIM41|75050|RI

CGREF1|52931|AP

RPS15A|34255|AA

DYRK1B|49847|ES

C7orf10|79305|AT

C7orf10|79306|AT

RPL7L1|76196|ES

CC2D2A|68806|AT

CC2D2A|68807|AT

TMPRSS13|18949|AT

MAZ|35938|AP

VPS51|16759|ES

MFF|57812|ES

ILVBL|48054|AP

ILVBL|48055|AP

TMEM205|47675|ES

SETBP1|45324|AT

SAR1B|73415|AP

QRICH1|64819|AP

QRICH1|64820|AP

PMPCB|81174|AT

PMPCB|81176|AT

NAAA|69561|AT

OXNAD1|63645|AA

ENTPD6|58868|ES

RAPGEF1|87968|ES

DRAM2|4136|AD

CALHM2|13011|AT

CALHM2|13010|AT

NFIX|47903|AP

CAB39L|25885|AP

ZNF233|50310|AT

ADARB1|60865|ES

CREM|11229|AP

KCNN3|7840|AP

MYO1B|56607|ES

DMPK|50522|ES

MAGED2|89250|RI

ITGA7|22211|AP

SRSF2|43660|RI

SEPT4|42688|AP

VWA5A|19211|AT

VWA5A|19212|AT

DMKN|49206|RI

MGAT5B|43698|ES

NUBP2|33138|ES

SBNO2|46390|AP

HDAC5|41773|AP

RPS5|52443|ES

GABARAP|38867|AD

PANK1|12493|AP

PANK1|12494|AP

LETMD1|21757|ES

MAP3K12|22062|AP

PARP9|66441|AT

PARP9|66442|AT

PUS10|53676|AT

PUS10|53675|AT

ERBB2IP|72261|ES

NUP62|51127|RI

POMGNT1|2783|AP

SETBP1|45325|AT

SLC12A4|37153|AP

ZNF233|50311|AT

KCNIP3|54503|AP

KLC4|76229|RI

YBEY|60914|ES

KCNIP3|54502|AP

FAM86A|33883|ES

NDRG2|26484|AP

ANKRD11|38082|ES

IL18BP|17481|RI

ZDHHC11|71449|AT

HPS1|12761|AT

HPS1|12760|AT

TRIP10|47079|AA

WIPF1|56031|AT

POMGNT1|2781|AP

EXD2|28129|AP

C14orf79|29590|ES

SMPDL3A|77390|ES

SYTL4|89603|AT

ZNF280D|30780|ES

RABL2B|62925|ES

TAF12|1389|AD

HPS5|14596|AD

MCF2L2|67797|AT

RHBDD2|80128|ES

MAGED4B|89152|AA

HDAC5|41772|AP

HDAC11|63476|AP

SEC16A|88178|ES

MTX2|56124|ES

GPBP1L1|2769|ES

SYTL4|89602|AT

SMUG1|22125|RI

IMMT|54414|AA

ACLY|40960|ES

TROVE2|9261|ES

TANGO2|61124|ES

PCK2|26827|AD

MEF2A|32717|ME

AXIN1|32866|ES

NIPBL|71817|AT

NIPBL|71818|AT

ATP5J2|80642|AT

ZNF843|36252|AT

ZNF843|36253|AT

MICAL1|77197|AP

MICAL1|77198|AP

FIP1L1|69313|ES

KLHL24|67805|AT

KLHL24|67804|AT

BIN1|55198|ES

ENTPD6|58866|ES

FGFR1|83430|ES

MTA3|53362|AP

PTBP1|46318|ES

HNRNPA2B1|79039|ES

CLK3|31725|AP

CLK3|31726|AP

NUMA1|17515|ES

CAB39L|25883|AP

PPIE|1902|AT

UBE2D4|79371|AT

UBE2D4|79369|AT

CCDC90B|18089|AD

LARP1B|70566|AT

WIPF1|56034|AT

GGA1|62120|AP

GGA1|62121|AP

MAPK7|39719|AP

TENC1|21924|AP

NME4|32875|AP

CCSER2|12406|AP

C1orf86|105959|ES

ZNF568|49433|AT

SUPT3H|76397|ES

DHX30|64544|ES

KATNAL2|45429|AT

APBB2|69096|AP

WWP2|37313|AP

TMEM177|55137|AD

WWP2|37311|AP

SCD5|69715|AT

LLGL2|43460|AT

LLGL2|43461|AT

PPIL3|56762|ES

CAPN3|30148|AP

NUDT18|82937|RI

LAMTOR3|70083|ES

ZNF396|45167|AT

ZNF675|48820|AT

JOSD2|51203|AP

JOSD2|51204|AP

KATNAL2|45430|AT

KAT6B|12264|AD

CALHM2|13012|AA

FAM131A|67936|ES

THAP3|486|AT

THAP3|487|AT

RNF213|44046|AT

DNM2|47588|ME

CEPT1|4140|AP

PPIE|1901|AT

MXRA7|43613|ES

SNRNP27|53861|RI

RCC2|854|AP

NPAS3|27143|AT

NPAS3|27142|AT

ZMIZ1|12300|ES

ABCF2|82356|AT

ABCF2|82355|AT

S100A13|7733|AP

PTPRC|9320|AT

FAH|32179|RI

ARSG|43135|AP

DYRK1A|60579|AA

FAM92A1|84519|AT

NRG3|12388|ES

BFAR|34098|ES

CDIP1|33755|AP

NME4|32879|ES

NIN|27493|AT

STK32A|73956|RI

SH3KBP1|88642|AP

CHTF8|37256|AP

CHTF8|37257|AP

KIAA1217|10993|AP

ATF7|22095|AT

COA1|79330|AT

ATG4D|47539|ES

ZNF131|71925|RI

TSEN2|63435|AD

C11orf68|16949|AA

BOD1L1|68797|ES

NRP2|57111|AD

ZNF396|45166|AT

CNNM1|12770|ES

COMT|61098|AP

ZBTB45|52478|AP

ZBTB45|52477|AP

SEC16A|88181|AA

UBE2I|33057|AD

HCFC1|90541|AA

OXNAD1|63638|AT

BIN1|55184|ES

ENTPD1|12653|AP

ABHD6|65430|ES

MGRN1|33784|ES

ABI1|11032|ES

HNRNPL|49697|AP

HNRNPL|49698|AP

MAPK7|39718|AP

RBM42|49227|ES

KIAA1958|87250|AT

KIAA1958|87249|AT

RAB11FIP1|83366|ES

KIAA0930|62646|AP

EXTL3|83221|AP

EXTL3|83222|AP

GSTT1|61369|ES

PCBP2|22051|ES

ANKRD13A|24394|AP

TSC22D3|89837|AP

POC1B|23621|AP

BTBD3|58699|ES

FBXO38|73981|ES

PAK4|49764|ES

PMS1|56543|AT

PMS1|56544|AT

DYNLT3|88789|AT

DYNLT3|88790|AT

ADAMTSL4|7484|AP

LRRC23|19997|AP

LRRC23|19994|AP

KIAA0930|62645|AP

FAM65A|37083|AP

TAF1D|18315|AA

PTPRC|9321|AT

DHPS|47832|ES

SCD5|69716|AT

UQCRQ|73319|AD

MOV10|4221|AP

ZKSCAN7|64365|AT

ZKSCAN7|64364|AT

KEAP1|47524|AP

POLR1D|25544|AT

POLR1D|25545|AT

R3HCC1L|12759|ES

FEZ2|53198|ES

C19orf82|47383|ES

NECAB3|102496|ES

POGK|8823|RI

OSBPL9|2979|ES

RAD54B|84553|AT

CEP63|66874|AP

UNK|43509|AT

HERC4|11913|AT

HERC4|11914|AT

PHLDB1|19035|AP

APLP2|19479|ES

EXD2|28128|AP

CD47|66015|ES

MIF4GD|43426|ES

STRAP|20589|ES

FNTB|27915|AP

FNTB|27914|AP

PPHLN1|21227|ES

ELMOD3|54216|ES

TBC1D14|68730|AP

GGA3|43410|ES

MSL1|40842|AT

MSL1|40843|AT

TMSB15B|89790|AD

TSC22D3|89836|AP

RPS21|60075|AA

ODF2|87755|AT

ODF2|87754|AT

ARSG|43136|AP

RAD54B|84556|AT

RNF167|38611|RI

KEAP1|47525|AP

NCBP2|68268|AD

MANBAL|59340|ES

ERCC1|50441|AT

ERCC1|50442|AT

COMT|61097|AP

SLC41A3|66564|AP

ETV7|75972|AT

FKBP5|75918|AT

FKBP5|75919|AT

PXN|24750|AA

SNRPN|29702|AA

ULK4|64260|AT

CAMK2G|12249|ES

ARAP1|17640|AP

HM13|58889|AD

SYDE1|48050|AP

SYDE1|48049|AP

MAN2A2|32507|AP

APOBEC3G|62277|AP

APOBEC3G|62278|AP

NLRX1|19130|AD

MCF2L2|67796|AT

RHBDL2|1859|AP

WDR6|64804|ES

FDPS|8059|ES

SYT15|11435|AT

SYT15|11433|AT

DGUOK|54011|ES

FEZ1|19295|AT

FEZ1|19294|AT

BCAS3|94643|ES

HKR1|49493|ES

POC1B|23620|AP

SLC9B2|70167|AP

RPL30|84639|ES

TMEM91|50043|AP

PKMYT1|33327|AT

PKMYT1|33328|AT

UCK2|8812|AP

UCK2|8811|AP

TFAP2C|59884|AP

MOV10|4222|AP

EVC|68699|AT

CITED1|89453|ES

PDSS2|77129|AT

PDSS2|77130|AT

TAF1D|18313|RI

GEMIN7|50396|ES

C12orf76|24403|AT

AP3D1|46577|AA

RPAIN|38681|ES

UNK|43507|AT

PMPCB|81177|RI

CTNNB1|64250|RI

RPAIN|38679|ES

PCSK5|86634|AT

CSMD2|1690|AT

TYR|260605|ES

PLEKHB2|55373|ES

RGS12|68643|ES

ATXN2L|35839|AA

TRAPPC2L|38047|ES

MTMR14|63113|ES

SOX15|39008|AT

SOX15|39009|AT

MAP2|57225|ES

ADAMTSL4|7485|AP

MAP7D3|90197|AT

MAP7D3|90198|AT

ITGB3|42068|AT

ASNSD1|56510|AA

MRS2|75506|AT

MRS2|75507|AT

RPS15A|34258|ES

RBM4|17096|ES

PEX26|61022|AT

PEX26|61021|AT

ZFC3H1|23406|RI

LIMK1|80060|AP

WDR53|68249|ES

S100A4|7714|ES

INCENP|16337|ES

APBB2|69095|AP

TMEM175|68431|ES

MORN1|252|AT

DCTN2|22644|ES

ACOT7|391|AP

SCAF11|21317|AT

PCBD2|73438|AT

PCBD2|73439|AT

HSD17B10|89224|ES

EEF1B2|57143|RI

CDIP1|33753|AP

GSTM1|4064|AT

GSTM1|4065|AT

GSTK1|82080|AD

RNASEH2B|25926|AT

RNASEH2B|25927|AT

RHBDL2|1860|AP

SYNE3|29170|AT

SYNE3|29171|AT

PCBP2|22052|ES

HNRNPA1|301521|ES

PAK4|49765|ES

SLC35A2|89036|AA

DMPK|50527|ES

ATXN2L|35847|AA

RPL17|94829|AA

ZNF263|33507|AP

ZNF263|33506|AP

PIK3C3|45316|AT

FHL2|54832|ES

IL18BP|17471|AT

IL18BP|17473|AT

MEIS1|53805|AP

HHAT|9699|ES

VGLL4|63394|AP

RWDD3|3828|ES

IFRD1|81445|AP

RNF213|44047|AT

EXOSC8|25656|ES

ST3GAL6|65789|AP

C17orf70|44129|AD

PARD3|11207|AT

CCDC120|89057|AP

CAMKK2|24853|ES

ZNF280D|30779|ES

PLA2R1|55711|AT

MOV10|4225|ES

HDAC11|63474|AP

CRYZ|3467|ES

PIK3C3|45317|AT

NME4|32873|AP

EPS15L1|48158|AT

SHF|30416|ES

PAX3|95774|RI

PPP1R1A|22194|AT

TTC7A|53483|AP

NADK|228|AP

CPNE1|59205|AD

MBD1|45514|AA

VWA9|31223|ES

FARP1|26163|AP

FARP1|26162|AP

RAB34|39953|AP

RAB34|39954|AP

ASPG|29539|ES

LRRFIP1|58133|ES

DPP6|82422|AP

RRN3|34141|ES

SCAF11|21319|AT

PPP2R5C|29317|AT

SMIM19|83735|AP

SLC41A3|66565|AP

FAM195B|44172|RI

GAS8|38205|AA

NSMCE4A|13332|AT

SERPING1|15871|ES

AP1S2|88572|AA

OPTN|10778|AA

RMDN1|84377|ES

UAP1|8750|ES

ANAPC7|24421|AT

ANAPC7|24422|AT

ZNF235|50304|AT

GPATCH2L|28543|AT

ACOT7|389|AP

CXorf40A|90314|AD

FLNB|65418|ES

ITGB1BP1|52625|ES

NINJ2|19605|AP

ZNF692|10568|AA

IFFO1|19883|ES

SPIN2A|89297|AP

ANXA11|12350|ES

PIK3CD|572|RI

TAF8|76166|AT

NIT1|8555|AT

NIT1|8556|AT

CAPN3|30147|AP

TMEM87A|30132|AP

TMEM87A|30133|AP

RPS9|211187|ES

RHBDF2|43588|AA

PLA2G15|37203|ES

RECQL5|43469|ES

NEMF|27445|AP

FAM63A|7539|AA

TTC7A|53482|AP

HPS4|61505|AA

CPNE1|59200|ES

TEAD2|50921|ES

SMARCAL1|57410|AP

SMARCAL1|57411|AP

KLC1|29465|AT

GLUL|9148|AD

PILRB|80936|RI

ST7|81553|AP

RCC2|853|AP

ABCA3|33265|AT

ABCA3|33266|AT

DNM2|47587|ES

HKR1|49492|ES

ARHGEF9|89302|AP

ZNF692|10557|RI

ETV7|75971|AT

ZNF564|47805|ES

NPIPA8|121053|ME

MTMR2|92805|ME

TNRC18|78664|ES

DUSP22|75130|RI

IMMP1L|14816|ES

FBXO7|61930|AP

FBXO7|61931|AP

SH3KBP1|88643|AP

HNRNPA1|22147|ES

UBTF|41827|AP

ZNF414|47241|AT

ZNF414|47240|AT

C20orf196|58656|AT

C20orf196|58657|AT

ARHGEF9|89301|AP

PEX13|53682|AT

PEX13|53681|AT

COG4|37401|ES

SLC44A2|47558|AP

IMMP1L|14819|ES

SRSF2|43666|ES

HDAC6|98334|AD

CABIN1|61388|AP

GRAMD1A|49011|AT

GRAMD1A|49010|AT

ABHD11|80028|ES

EPB41L5|55146|AT

EPB41L5|55145|AT

MFF|57816|ES

ZNF131|71918|AP

CFLAR|56791|AT

GGA3|43399|RI

SESN1|77155|AP

LTBP4|49935|ES

GRB2|43439|AP

ABHD14B|65144|ES

FDFT1|82643|AP

PEX11A|32438|ES

MAPKAP1|87586|ES

C5orf45|74970|ES

WDR52|66200|AT

WDR52|66199|AT

MAZ|35937|AP

SSBP4|48431|ES

RPP14|65434|AD

MTA2|16357|AP

GRB2|43438|AP

PTK2|85302|RI

SLC9B2|70169|AP

NMRAL1|33737|AD

RCC1|1386|AP

RCC1|1387|AP

MAGED4B|89151|AA

FAM65A|37081|AP

NPRL3|32804|ES

TGIF1|44502|AP

CACNB3|21468|AP

TTC12|18773|AT

SPECC1|39790|AT

SPECC1|39792|AT

IER2|47929|AP

IER2|47930|AP

BRE|95458|ME

DPP6|82421|AP

RASEF|86676|AT

RASEF|86677|AT

SLC3A1|53419|AP

EVC|68700|AT

LRRC37A|42022|AP

LRRC37A|42021|AP

UBTF|41830|AP

CACNB3|21469|AP

LDB3|12423|ES

NPIPA8|34239|ES

SORBS3|83014|AP

SORBS3|83015|AP

TACC1|83449|ES

MROH6|85426|ES

GPATCH2L|28542|AT

SLC22A18|13934|AP

MADD|15716|ES

EIF2A|67287|ES

NDUFA7|47217|AT

AKT1|29569|AD

UBTF|41831|AP

WIZ|48091|ES

NUP214|87926|AA

NUDT22|16590|RI

PRKDC|83791|ES

PLA2R1|55710|AT

FBXO18|10670|AP

FLNB|65417|AD

ATXN2L|35838|RI

TBC1D16|44014|AA

SSBP3|3144|ES

ZNF783|82182|AT

ZNF783|82183|AT

SLMO1|44686|AP

SAA2|14577|AT

IFRD1|81446|AP

ZNF174|33572|AT

ZNF174|33571|AT

TMEM107|39123|AA

FAM114A1|69037|ES

ZNF213|33458|AD

PSMG3|78590|AP

GSTT1|61384|ES

FLOT2|40004|ES

SHF|30407|AP

TYMP|62850|AA

ULK4|64258|AT

FAM60A|20982|AP

TRAFD1|24580|AD

FLJ27365|62680|AT

FLJ27365|62681|AT

ATP5J2|80640|AT

ASCC2|61681|ES

ZNF814|95404|ES

OAZ1|46599|ES

TBXAS1|81965|AP

FAM60A|20983|AP

TFDP1|26384|AP

PRKAG1|21508|ES

RPS3|17843|ES

GSTT1|61375|ES

MAST4|72281|AT

HRAS|13685|ES

TM4SF19|68239|AT

GSTO2|13050|AT

GSTO2|13051|AT

DNAJC14|22260|AP

DNAJC14|22262|AP

NDUFA7|47215|AT

ZNF568|49432|AT

ZNF771|36105|AT

SCO2|62847|AP

TMSB15B|89789|RI

PRKRIP1|97666|ES

ICA1|78794|ES

ORMDL1|56538|AA

COX4I1|156376|ES

OXLD1|44145|AD

TMEM62|30215|ES

YY1AP1|8104|AA

ABCA2|88252|RI

GLS|56589|AT

GLS|56590|AT

PPP1R1A|22195|AT

TBXAS1|81964|AP

TMUB2|41810|ES

SLC3A1|53420|AP

RHOF|24897|AT

RHOF|24898|AT

HPS5|14595|ES

ZNF185|90397|AP

GOLGA4|63982|ES

CIRBP|46421|RI

DNAH2|39052|AT

SLC22A18|13935|AP

LY6E|157706|ME

ST7|81563|ES

CITED1|89452|AP

NFKBIB|49718|RI

APBB3|73661|RI

RNF43|42674|AD

ZNF771|36106|AT

WDR4|60760|AT

WDR4|60761|AT

MRPS12|49736|RI

TAZ|90586|ES

TTLL3|63211|ES

TMEM194B|56568|AP

NAT9|43289|RI

SCO2|62846|AP

FOXP1|65608|ES

EVL|29240|AP

ADCY6|21465|AP

ADCY6|21464|AP

HIPK2|81963|AA

NMRAL1|33727|AP

JARID2|75408|AP

FAM184A|77361|AT

DPP9|46826|ES

ZNF7|85667|ES

PARP3|65116|ES

HDAC8|89464|AT

AK5|3523|AP

NPIPB5|35571|AD

SMIM7|48186|AA

FNIP2|70999|AP

CPNE1|59204|ES

KTN1|27637|ES

FMO5|7367|AT

KIAA1217|10995|AP

MC1R|38164|RI

HPS1|12762|ES

CD74|152981|ES

ENY2|84894|AD

TRIM41|75051|RI

PPM1K|69887|ES

SIK3|18878|ES

MEIS1|53804|AP

SLC44A2|47559|AP

DCTD|71235|ES

CGREF1|52930|AP

GTPBP8|66125|AT

GTPBP8|66126|AT

DDX20|4190|RI

INO80E|36018|AA

SIGIRR|13650|AP

PHF17|70581|AT

PHF17|70582|AT

TMEM177|55138|AD

NPHP1|54927|AT

NPHP1|54928|AT

FAM184A|77360|AT

POLL|12891|ES

RPS6KA1|1281|AP

PON2|80535|AA

VEGFA|76318|AP

VEGFA|76319|AP

GAS2L1|61591|RI

EPC2|55538|AT

EPC2|55539|AT

LRR1|27427|ES

RAMP2|41120|AP

RAMP2|41121|AP

SYNJ2|78242|AP

HDAC10|62806|ES

PCNXL2|10324|AT

HNRNPA1|212645|ES

ANKLE2|25296|AP

CD97|47969|ES

C15orf39|31839|AD

HHLA3|3402|ES

UBXN11|101233|ES

TACC1|83434|AP

TTC12|18774|AT

HELZ2|60135|AP

GRK4|68625|AT

GRK4|68624|AT

MRPL55|10177|AD

HHAT|9689|AP

RUSC2|86251|AP

ADCY1|79595|AT

ADCY1|79596|AT

TAZ|90595|ES

SWI5|87732|AA

DNAJC2|81187|AT

DNAJC2|81185|AT

ZMYND12|2070|ES

DGUOK|54006|ES

PLEKHA5|20648|AT

C1orf213|1054|RI

ATP5C1|10726|ES

PRPF39|27398|ES

NEU3|17816|ES

RUSC2|86252|AP

RAD51B|28110|AT

POLG|32420|AD

NPIPB4|35511|ES

ZNF692|10563|RI

STXBP6|27041|ES

CCPG1|30726|RI

CSAD|21943|AP

RAD52|19635|AP

RAD52|19633|AP

C11orf74|15441|ES

COX16|28178|ES

TMEM91|50053|RI

MXRA7|43608|AP

SLC25A29|29257|AA

PTK2|85317|ES

MTIF3|25537|AP

UBXN11|1259|ES

IDUA|68441|RI

IDNK|86687|ES

BCL2L1|58902|AA

DYRK1B|49845|RI

PCNXL2|10323|AT

SLC3A1|53425|AT

HSPBP1|52052|AP

NPIPB4|35509|ES

BCAT2|50815|ES

U2AF1L4|49280|AA

TCF12|30784|AP

TNPO1|72474|AP

GMFG|49768|RI

RPLP0|24733|ES

CRTC1|48504|ES

RPS3|17853|ES

NPM2|123608|ES

PGAP2|14025|ES

BSCL2|16403|AP

RAD51D|40263|ES

MORF4L2|89765|AP

FAM160B2|82936|ES

ACP5|47747|AP

ACP5|47745|AP

DNASE1L1|90576|AD

ARMC5|36262|RI

ZSWIM7|39393|RI

MVP|35966|AA

MTIF3|25536|AP

FAM45A|13256|AA

PDE4D|72142|AP

CIRBP|46423|RI

GSTM2|4057|AT

KIAA0319L|1718|AP

GANAB|16378|ES

NGLY1|63754|ES

NSMCE4A|13331|AT

SEC31A|69730|ES

SFI1|61868|ES

POLR2J2|81128|AA

C14orf80|29662|ES

S100A13|7731|AP

ACSBG1|32055|AP

VWA9|31228|AD

FAM3A|90639|ES

OXNAD1|63639|AT

KIAA1549|81938|AA

IFIT3|12488|AP

FRG1B|58883|ES

SUPT7L|53038|ES

PHF21A|15538|ES

CCDC120|89058|AP

ULK2|39775|AT

ULK2|39776|AT

ATXN7L1|81308|AT

SHMT1|39617|ES

BAIAP2|44102|ES

SNCA|69932|AD

CELF1|15765|AA

FAM102A|87690|AP

FAM102A|87691|AP

KIAA0195|43443|AP

RHOC|4236|ES

MAP2|57227|ES

SLC6A11|63374|AT

SLC6A11|63375|AT

U2AF1L4|49276|ES

C6orf1|75778|AD

SLC35C1|15509|RI

RPRD2|7472|ES

WSB1|39835|RI

PLCB3|16608|AT

PLCB3|16609|AT

P4HA2|73257|ME

CALD1|81856|AP

PFKFB2|9613|AP

STAU2|84152|AP

COA1|79335|AT

ZNF384|19923|ES

PSTPIP1|31973|ES

ZFP41|85401|AT

ACSBG1|32056|AP

MS4A6A|16057|RI

DCTD|71250|ES

MTMR14|63110|ES

TRAIP|64922|AT

TRAIP|64923|AT

ZNF726|48830|AT

FAM19A5|62733|AP

BFAR|34095|ES

RPRD1A|45199|AT

RPRD1A|45201|AT

ZNF280D|30771|AT

SYNJ2|78244|AP

GBGT1|88022|ES

SEPT2|58372|ES

PCDHA9|73785|AT

PCDHA9|73784|AT

ADCK5|85594|ES

SOS1|53315|ES

DGUOK|54012|ES

PDE4D|72140|AP

SULT1C2|54877|AA

KIAA0195|43444|AP

GNAS|60003|ES

ACAP1|38921|AP

TMEM14B|75312|AT

OGFOD2|25005|AP

OGFOD2|25006|AP

NMRAL1|33728|AP

PPIP5K1|30267|ES

SUN2|62262|ES

ACAN|32395|AT

PDLIM5|69971|AT

COASY|41069|AA

TMSB15B|89787|AT

TMEM194B|56569|AP

PSAP|12067|AA

NPM2|127833|ES

FDPS|8074|AA

UBXN11|1263|ES

APOC1|50371|ES

UQCR11|46527|AT

UQCR11|46528|AT

RBMS1|55728|ES

ZNF692|10567|AA

DNAH2|39053|AT

VWA9|31227|AD

ZNF276|38135|AP

ZNF395|83210|AP

GEMIN7|50397|ES

ZNF280D|30770|AT

DUT|30485|AP

EFEMP2|16931|RI

HNRNPUL1|50034|AA

ARHGAP10|70799|ES

CLASRP|50392|AD

SLC25A39|41846|AA

DMKN|49205|RI

UQCC1|59085|AT

FLT3LG|50949|AA

CDK11A|213|AD

ZNF462|87145|AA

FAM228B|52816|ES

HDHD2|45441|ES

SESN1|77156|AP

TRIP12|57856|ES

GEMIN6|53289|ES

NLRP1|38720|AT

SNAPC2|47195|AP

SNAPC2|47196|AP

CENPK|72213|AT

WDR54|54055|ES

CENPK|72212|AT

RPS9|211191|ES

CYB561A3|16163|RI

PTK2B|83150|AP

ETV4|41709|AP

ZNF446|52471|AD

CSF2RA|88369|ES

TVP23A|33992|AT

CCSER2|12405|AP

TMCO6|73694|RI

NBPF10|4452|ES

SIGIRR|13651|AP

PLEKHA4|50820|ES

TRAF3IP3|9681|AT

BCKDHA|50062|AP

BCKDHA|50063|AP

GANC|30141|AT

PDZD7|12863|AT

ABHD14B|65143|ES

C21orf67|60854|AT

C21orf67|60855|AT

ATP5J|60270|AD

TMUB2|41789|ES

NPIPB4|35514|AD

KLHDC4|37947|AT

CCT7|53961|ES

ZYX|82115|AP

CBX5|22140|AP

CBX5|22139|AP

ATXN7L1|81309|AT

MYO1C|38309|AP

ANKRD29|44842|AT

IFIT3|12489|AP

ABHD14B|65146|AA

MIA|95218|AP

KLHL5|69040|AT

TRA2B|68039|ES

TRIM3|14129|AD

ADAM12|13452|AT

ADAM12|13453|AT

TMEM161A|48587|ES

SHF|30409|AP

MLH3|28469|ES

AAK1|53858|AT

PRELID2|73898|ES

CAMLG|73423|ES

MFSD9|54806|AA

FXN|86527|AT

FXN|86528|AT

SAT2|39032|AD

FBLIM1|771|AP

ELOVL5|76493|AT

ELOVL5|76492|AT

UGGT2|26131|AT

ST3GAL6|65791|AP

F11R|8517|AP

POLM|79449|AD

TRAPPC3|1754|AD

PILRB|80935|ES

DNAJC21|71746|ES

AP2M1|67841|ES

EMC8|37897|ES

EBPL|25911|ES

EPS15L1|48153|AT

UPP1|79639|ES

ADAL|30232|AT

ADAL|30234|AT

TVP23A|33993|AT

CYTH1|43887|AP

ACACA|40505|AP

BCLAF1|77906|ES

C17orf49|38824|ES

PSRC1|119798|AD

CEP164|18915|ES

CNOT4|81884|AT

ANKLE2|25295|AP

C11orf80|17129|ES

DPH2|2503|AD

PTAR1|86545|AT

PTAR1|86546|AT

ATXN2L|35860|AD

TPP2|26212|ES

TFAP2C|59883|AP

LPXN|16009|AP

BAX|50836|RI

CYP2R1|14477|AP

LARP1B|70567|AT

SMN1|72422|AA

LAS1L|89321|ES

PDLIM7|74777|AT

RPAIN|38691|ES

ZNF397|45149|AD

IFI35|41177|AD

FARP2|58381|AT

FARP2|58380|AT

STK32C|13485|AP

ATAD3A|175|AP

ATAD3A|174|AP

AGAP1|58088|ES

IKBIP|23865|AT

IKBIP|23864|AT

SRSF3|75985|ES

TMPRSS13|18947|AT

GLG1|37565|RI

POLR2J3|81117|AA

FASTK|82342|AD

ERCC1|50447|AD

TMEM107|39107|RI

DHRS4L2|26795|AP

DHRS4L2|26796|AP

UBE2I|33061|ES

FDFT1|82638|AP

LAT2|80064|AT

LAT2|80065|AT

DHRSX|88403|ES

SLC37A2|19257|ES

TCF12|30783|AP

NAT9|43297|ES

CDCA3|19987|RI

ZNF317|47268|AA

DOCK7|3256|ES

G6PC3|41765|ES

DGUOK|54008|ES

CCDC130|47932|AP

CCDC130|47933|AP

CATSPERD|46924|AT

CATSPERD|46925|AT

PIWIL4|18357|AP

CCDC90B|18069|AD

MST1|64899|RI

SLC11A2|21729|AT

SLC11A2|21730|AT

PORCN|88982|ES

STAU1|59738|ES

USP6NL|10750|AT

USP6NL|10751|AT

MORN1|255|ES

PPM1M|65193|ES

R3HDM4|46352|ES

ACAN|32396|AT

CNDP2|45816|AA

SLC3A1|53421|AT

MDFIC|81513|AT

MDFIC|81514|AT

NPIPB5|35566|ES

ZNF331|51723|AP

C14orf80|29661|ES

APBB3|73676|ES

SGSM2|38394|ES

NLRP1|38719|AT

C9orf3|86945|AT

ABCC5|67814|AT

RPP38|10863|ES

B3GALNT2|10352|AT

B3GALNT2|10353|AT

ZNF397|45143|AT

TSGA10|54659|AP

AK5|3524|AP

IL17RC|63259|ES

NDRG2|26485|AP

MTCH1|76000|AD

CCNT2|55407|RI

CYTH1|43888|AP

ALDH3B1|17271|AP

ABCC5|67815|AT

FAM53B|91871|ES

GSTA4|76482|ES

FBLIM1|769|AP

RPS20|83888|AA

ASTN2|87368|AT

PIGV|1302|AD

ASTN2|87369|AT

SORBS2|71383|ES

DDIT3|22625|RI

CIRBP|46426|RI

BATF2|16723|AP

GNPDA1|73865|ES

AP1S2|88569|AT

AP1S2|88571|AT

HDAC8|89459|AT

ZNF207|40206|ES

CHRNA3|32113|AT

BRD8|73509|ES

HSF4|36942|AA

RPSA|64183|ES

ATXN2L|35846|ES

SERBP1|3355|AA

C7orf31|79030|AD

C5orf45|74971|ES

ZNF680|79826|AT

ZNF680|79827|AT

CPSF4|80629|AP

SRRM1|1128|ES

TMEM218|19279|AA

CPSF4|80630|AP

PACRGL|68885|ES

ANGEL1|28558|AP

GSTM2|4061|AT

CBWD2|55054|ES

LGALS3|27618|AT

LGALS3|27617|AT

ANGEL1|28557|AP

C1orf213|1056|RI

ATOX1|74174|AP

PHF19|87400|AP

MTHFSD|37920|ES

LPXN|16010|AP

MRPL55|10084|AD

ZNF559|47272|AP

MTA1|29643|ES

NOX5|31378|ES

KIAA0430|34174|AA

CD44|14976|ES

POLL|12887|AD

STK25|58390|AD

CDK18|9553|AD

BIN1|55192|ES

STK11IP|57739|AD

TRIM5|14072|RI

MITF|65590|AA

NPIPB3|34443|AP

TMEM237|56846|AP

LONRF1|82746|AP

ZNF257|48803|AT

RILP|38360|ES

ZNF257|48804|AT

PTK2B|83149|AP

C14orf80|29657|AP

TAF8|76167|AT

PSPC1|25404|AT

TEAD4|19741|AP

EIF1AD|16960|AA

ZSCAN30|45155|AT

ATP9B|46234|ES

AMDHD2|93845|ES

ZSWIM8|12234|AA

UCHL5|9238|AP

OGFOD2|25009|ES

ABCC5|67827|RI

CAPN3|30160|ES

C1orf63|1147|ES

RIOK2|72877|AT

RIOK2|72878|AT

TAF1D|18318|RI

SDCCAG3|88163|ES

HSF1|85559|ES

C19orf25|46502|AT

C19orf25|46503|AT

EIF1AD|16961|AA

SLC11A2|21731|RI

AP1S3|57760|AT

IKBKE|9584|ES

TEAD2|50920|AD

ALDH3B1|17270|AP

SLC7A6|37208|RI

RANBP3|46967|ES

PHF19|87399|AP

ACACA|40506|AP

ZNF771|36108|AA

TMSB15B|89788|AT

AURKA|59846|ES

ATOX1|74184|ES

PCGF2|40584|AP

SMARCC2|22391|AD

FDFT1|82657|ES

ZNF576|50227|AD

CAMK1D|10772|AT

CAMK1D|10773|AT

ZBED5|14401|AA

R3HDM1|55443|ES

BCL6|68081|AP

FHIT|65492|AA

MFF|57815|ES

ZNF283|50245|AT

SIK3|18875|AA

TMUB2|41817|AA

FAM122C|90163|AT

PARD3|11208|AT

DUT|30484|AP

LARP6|31442|AT

LARP6|31443|AT

STAU2|84153|AP

SLC13A3|59696|AT

ATXN2L|35851|ES

CAPS|46956|AD

ZNF655|80690|ES

IL12RB1|48393|AT

IL12RB1|48392|AT

GUSB|79855|ES

SLC6A6|63528|ES

MTAP|85995|AT

MTAP|85996|AT

RNF167|38614|AD

ARPP19|30692|RI

SEC31A|69729|AD

VEGFA|76330|ES

ATP5A1|300061|ES

GPR56|36588|ES

ASB9|88545|AD

UQCC1|59083|AT

RBM14-RBM4|17102|ES

CCDC106|52132|AA

ST7L|4210|ES

PMM2|33931|ES

CAPN3|30158|ES

TMEM175|68428|AA

ECI2|75226|AA

N4BP2L1|25590|ME

RNF220|2559|AD

MICALL2|78570|AP

DCAF6|8886|ES

STK36|57555|AP

ANKRD30B|44760|AT

ANKRD30B|44762|AT

MAP4K4|54763|AA

PTPRH|52047|ES

HPS5|14594|AD

PLEKHA5|20646|AT

PLD3|49893|ES

MUTYH|2675|ES

UNKL|33077|AP

CAMTA2|38639|AA

PSRC1|4001|RI

MYO19|40489|ES

PML|31633|AA

TMEM175|68433|ES

CCDC34|14752|AT

CCDC34|14751|AT

MAF|37687|RI

TMEM219|35991|AP

FBXO4|71889|ES

RPL36|46861|RI

SRSF7|53279|RI

SPATA6L|85752|AT

GIGYF2|58024|ES

HM13|58892|ES

MX1|60664|AP

SP100|57895|AT

SLC38A6|27787|AA

MOV10|4226|AA

NUDT6|70523|AP

DGUOK|54007|ES

BTD|63622|AD

TMEM219|35990|AP

PDLIM7|74778|AT

CACNB3|21476|ES

G3BP1|74194|AA

SNRPD2|50511|ES

DCAF11|26839|ES

BBIP1|13094|ES

PSMD3|196289|ES

ING4|19912|AA

ATP5J|60267|AD

SNX13|78878|AT

SNX13|78880|AT

PTGER3|3412|AT

LUC7L|32845|AD

SRRM1|1133|ES

EPB41L1|59273|ES

SSH1|24258|ES

WIBG|22287|AP

MFF|57810|ES

AMPD2|4049|ES

PLA2G6|62213|AA

ATP6V1E2|53462|AP

AMMECR1L|55250|AP

AMMECR1L|55251|AP

CAMLG|73424|ES

PSPC1|25402|AT

PRDM2|725|ES

LCLAT1|53115|AT

LCLAT1|53116|AT

PIGF|53467|ES

UBR4|872|AP

AKT1|29567|RI

PTCD2|72469|AD

BCL6|68079|AP

SETMAR|62991|AT

SETMAR|62992|AT

AKAP2|87177|AP

FILIP1L|65812|AT

EVC|99903|ES

CTAGE5|27371|AP

IFT46|19025|ES

UBXN11|1255|ES

POLL|12886|RI

IFI27|29086|ES

PDXK|192507|ES

OXLD1|44143|AD

LRRC37B|40172|ES

FAU|16772|AA

CCDC159|47686|AD

ELMO2|59686|ES

NFYB|24094|AP

ABI1|11042|ES

NPIPB4|35513|RI

TMEM237|56847|AP

MARK3|29450|ES

NAV2|14697|ES

TMUB2|41823|ES

CLNS1A|17957|AT

CLNS1A|17956|AT

G3BP2|69549|AP

AJAP1|353|AT

RBMX|90220|RI

CUL1|82147|AP

ATXN2L|35854|RI

TCF7L2|13149|AA

AKAP6|27135|AT

CXorf40A|90308|AD

RPS9|211197|ES

SERF1B|72405|AT

BCAP29|81361|ES

RAB27A|30714|AP

AKAP6|27136|AT

THOC5|61613|ES

CIRBP|46427|AA

CLSTN1|576|ES

CBWD1|85690|ES

B3GAT3|16371|AA

WIBG|22288|AP

CPSF4|80635|AA

ST7|81554|AP

TMED4|79538|AT

TMED4|79539|AT

HNRNPD|69698|AT

HNRNPD|69699|AT

SH3YL1|52491|AP

ULK4|64257|AT

CENPI|89628|AP

CENPI|89629|AP

ZSCAN5A|52162|AP

BCCIP|13431|AT

FAM122C|90162|AT

FAM49B|85161|ES

ADK|12258|AP

RSU1|10880|RI

SMIM19|83734|AP

HDDC2|77424|ES

MTFR1L|1211|ME

NUTM2F|86930|AT

AFMID|43791|AD

AJAP1|352|AT

MFSD12|388179|ES

HNRNPA2B1|79037|ES

TMEM117|21283|ES

BDH1|68301|ES

NIN|27491|AT

GPSM1|88151|AP

ABLIM2|68744|AT

ABLIM2|68745|AT

PTCH1|86956|AP

F11R|8516|AP

ATG13|15592|ES

RPS11|50954|ES

CTBP2|13412|AP

ALG2|87057|ES

SH3PXD2A|13023|AP

MTA2|16358|AP

ATXN2L|35845|RI

REPIN1|82247|ES

WDPCP|53725|AP

TIAM2|78228|AP

THTPA|26763|AD

TMEM55B|26455|AD

MGRN1|33780|AA

KLHDC4|37951|AT

FAM49B|85160|ES

SLC2A11|61349|AA

MBD1|45521|ES

LRP2BP|71336|AP

BUD31|80623|AD

MICALL2|78571|AP

TIAM2|78232|AP

GNAI2|64968|AP

FBXO33|27381|ES

C5orf22|71633|ES

ST3GAL4|19394|AP

KRBOX4|88886|AT

APPL2|24134|AA

CCDC25|83179|ES

PPP2R5C|29319|AT

KIAA1429|84563|AT

KIAA1429|84564|AT

KIAA0319L|1719|AP

TMPRSS13|18948|AT

RPP30|12504|AT

RPP30|12505|AT

CREBZF|18137|RI

SERF1B|72403|AT

BTN3A2|75619|AA

ILK|14177|AA

SUMF2|79798|ES

FAM19A5|62732|AP

LTBP4|49931|ES

RNASE1|26469|ES

PACS2|29633|AP

TEFM|40127|RI

SH3BP5|63562|AP

FBLN5|28893|ES

S100A13|7732|AP

NOP2|19895|AD

ARHGEF7|26280|AP

ABCB6|57633|ES

PFDN5|22008|ES

GINS4|83515|AT

GINS4|83516|AT

DDX42|42993|ES

CHRNA3|32114|AT

ACTR8|65321|AP

ACTR8|65320|AP

STX16|59970|ES

ALKBH2|24276|AA

ORC3|76967|AA

SCMH1|2051|ES

PIAS2|45421|AT

PIAS2|45422|AT

ZYX|82114|AP

FAM110A|58470|ES

MANBAL|59344|ES

LTBP4|49930|ES

C14orf80|29660|AP

ELK1|88937|ES

EVA1A|54150|AP

CFLAR|56792|AT

TECR|564143|ES

ZBTB7B|7878|ES

ZNF235|50303|AT

SETMAR|62998|ES

UCHL5|9237|AP

COX11|42568|AA

ARHGEF7|26277|AP

HKR1|49489|ES

LETMD1|21747|ES

TRIM5|14078|AD

ZNF197|64375|AT

ZNF197|64376|AT

SMAD6|31294|AP

SMAD6|31295|AP

SLC2A8|87631|ES

AMBRA1|15576|ES

RPUSD3|63229|AD

ZNF554|46626|ES

RPL29|65167|RI

TTLL3|63205|AT

TTLL3|63206|AT

HAUS1|45389|ES

ATF7|22090|AP

RDM1|40348|AT

COA1|79339|AD

SEPT9|43723|AP

SPATA6L|85753|AT

MEF2B|48596|ES

BSCL2|16409|AD

ADK|12257|AP

ZNF76|75905|AA

BTN3A1|75662|AA

G3BP2|69548|AP

IKBIP|23866|ES

EBPL|25915|ES

NSG1|68674|RI

CYTH1|43891|ES

MACROD2|58716|AP

RDM1|40349|AT

PMEPA1|59948|AP

ANKRD10|26270|AT

ANKRD10|26271|AT

MAP2K7|47192|AA

HOMEZ|26723|AP

HOMEZ|26724|AP

ATG13|15594|ES

TMEM14C|75307|AA

OPTN|10781|ES

HCFC1R1|33353|AA

DNAJC19|67760|ES

DECR1|84407|ES

KREMEN1|61570|AD

NAF1|71008|AT

CASP10|56801|AT

POR|80136|RI

TRRAP|80596|AD

LRWD1|81100|AD

LZIC|586|AP

LZIC|587|AP

FKBP7|56168|ES

OSBPL1A|44877|AP

OSBPL1A|44876|AP

NSFL1C|58500|ES

NSMCE4A|13328|AP

MIB2|183|AP

GNAS|60006|ES

SNRPN|29705|ES

SIRT5|75391|AT

SIRT5|75393|AT

ZNF436|1050|AP

CASP10|56802|AT

PLCD4|57506|ES

PFDN5|22004|ES

PBX3|87592|ES

ZSCAN32|33548|RI

ACBD4|41946|AA

CTSB|82673|ES

NPIPB5|35570|RI

SPRTN|10277|RI

SCYL1|16860|AA

CLASP1|55177|ES

DYNLL1|24765|AA

CHMP2A|52483|AP

CYHR1|85614|AP

MACROD2|58715|AP

TMEM205|47656|RI

CYHR1|85612|AP

UBE2A|89957|ES

TEAD4|19740|AP

EVA1A|54148|AP

NUTM2F|86931|AT

TNFAIP2|29435|RI

ZNF519|44758|AT

ERBB3|22351|AP

NMNAT3|67040|ES

JKAMP|27751|AA

MDH2|117319|ES

LIMS2|55227|AP

BCL2L13|96061|ES

ZDHHC24|17066|ES

NNT|71963|AP

LTBP4|49937|ES

CD96|66049|AT

ZNF592|32326|ES

EPB41L1|59271|ES

PPHLN1|21228|ES

PLIN5|46809|AT

PLIN5|46808|AT

TP53I3|52810|ES

ACAP1|38918|AP

SGK1|77772|AP

NFYB|24093|AP

PLEC|85511|AP

FAM104B|89278|AT

FAM104B|89277|AT

ADAMTS13|88052|AD

GNAI2|64970|AP

CTDP1|46247|ES

OLFM2|47443|AP

MINK1|38598|ES

SMARCC2|22390|RI

CEP63|66882|ES

SCRIB|85500|ES

NOP2|19890|ES

OLFM2|47444|AP

CHMP2A|52481|AP

FLNA|90565|ES

BCCIP|13433|AT

COASY|41062|RI

NPIPB3|34444|AP

SAA2|14580|AT

C16orf95|37937|ES

DALRD3|64809|RI

FCHSD1|73817|ES

PFKM|21421|AD

EIF3M|14855|ES

TRIM16L|39639|ES

EXOC3|71442|AD

KLHL12|9423|AP

TRIM65|43525|ES

SCIMP|38666|AT

SCIMP|38667|AT

MGRN1|33781|ES

ZNF706|84743|ES

RPE|57242|ES

PPP2R4|87833|AP

SERF1A|72416|AT

MLTK|55999|AT

MLTK|55998|AT

MAN1C1|1192|AP

MAN1C1|1193|AP

MYL5|68388|ES

SPIN2B|89294|RI

CIZ1|87710|AP

MLPH|58115|ES

OGFOD2|25012|AA

TCF7L2|13156|ES

C19orf60|48492|AA

HEXIM2|156419|AD

OTUB1|16553|AP

UBR4|875|AP

ZNF584|52456|ES

TFDP1|26387|AD

RAB6A|17707|ME

DNASE1L3|65424|RI

AP5S1|58606|AD

RARG|21979|AP

RARG|21980|AP

HHLA3|3405|AD

TRPM6|86615|AT

NINJ2|19604|AP

MANSC1|20502|AP

SHF|30414|ES

ZNF131|71921|AP

PACRGL|68876|AT

PACRGL|68877|AT

COPS5|84071|ES

MFF|57807|ES

CPT1B|62867|AP

EVC|96676|ES

GALT|86202|ES

MRPL55|10147|ES

NSG1|68675|AD

STX16|59983|ES

TSPAN4|13789|AP

RPS5|52444|ES

ENPP2|85000|AP

UBP1|63867|ES

USP21|8566|ES

MFF|57809|ES

FLII|39592|AA

LUC7L|32841|RI

CIRBP|46424|RI

C3orf18|65073|ES

CBLB|66001|ES

ATOX1|116361|ES

EYA3|1369|ES

NMRK2|46756|AA

CCDC65|21489|AT

CCDC65|21490|AT

CSNK1A1|74047|ES

AMPD2|4043|AP

CCPG1|30723|AP

ZNF436|1051|AP

ABCC5|67819|RI

MAT2B|74448|AP

MAT2B|74449|AP

AAGAB|31308|AD

G3BP2|69550|ES

CD46|9663|ES

RPL34|100828|ES

EPOR|47690|AA

CXorf40A|90295|AP

CXorf40A|90298|AP

TPCN2|101162|ES

SRSF5|28161|AD

DTX3|22660|AA

MLLT10|10970|AT

MLLT10|10971|AT

MAN2A2|32509|AP

CYB5RL|3128|AT

CYB5RL|3129|AT

TVP23C|39356|AT

CCDC51|64651|ES

SYNJ2|78249|AD

MARK2|16540|ES

PFKM|21422|ES

UBA52|48469|AP

STX18|68685|AT

STX18|68684|AT

TMPO|23844|AT

CYB5R2|14208|ES

DYNC2LI1|53402|ES

MED6|28185|AA

TAZ|90601|AD

ARID1B|78237|ES

MORF4L2|89771|ES

CGREF1|52940|ES

KIAA0391|27217|ES

MAP4K1|49669|ES

RDH13|52001|AT

RDH13|52000|AT

ARPP19|30690|ES

ZSCAN5A|52163|AP

C12orf73|24075|ES

MPV17|52976|AA

OSBP2|61807|ES

PHYKPL|74853|AT

PHYKPL|74854|AT

SENP1|21408|AP

ARAP1|17639|AP

LRSAM1|87637|RI

MUTYH|2672|AA

DMKN|49167|ES

ZNF44|47789|AT

ANKHD1|73657|AA

IGFLR1|49261|ES

ZNF124|10514|AT

MANSC1|20503|AP

HERPUD1|36506|ES

GABARAP|38868|ES

C21orf2|60816|AD

HNRNPA2B1|79033|RI

DMTN|82930|ES

SRSF2|43661|RI

RNF7|67078|ES

BRD8|73507|ES

C12orf76|24406|AT

TRPM6|86616|AT

LDHD|37582|AA

RALGDS|88017|AA

TFDP1|26389|ES

PPP1R3B|82593|AP

OSGEP|26442|AD

HVCN1|24488|AP

RAP1GAP|992|ES

ZNF675|48822|AT

REEP5|72992|ES

HDAC6|89008|AP

TMPO|23845|AT

KANSL1|42011|AP

AIG1|77972|AT

CPNE1|59189|AA

NPIPB4|34757|RI

LYRM1|34404|AP

SH3YL1|52495|AP

CDKN2A|86004|AP

STAU1|59737|AA

SMIM12|1701|AD

ADPGK|31594|AD

ZNF581|52119|AP

KIAA0895|79246|AT

KRBOX4|88888|AT

REPIN1|82236|ES

CDK12|40649|AA

ANKRD27|48899|ES

PLA2G4C|50682|AD

UPRT|89523|ES

PCM1|82838|ES

NEIL2|82633|ES

WDPCP|53726|AP

RNPS1|33261|ES

EPOR|47689|RI

TSGA10|54661|AP

ZNF765|51718|AT

ZNF765|51715|AT

CCDC115|55334|ES

GPN1|53035|ES

CCDC41|23727|AT

CCDC41|23728|AT

DNAJA4|32067|AP

NEK6|87526|AP

TRIM16|39367|ES

NSMCE4A|13329|AP

CEP78|86656|AT

CEP78|86655|AT

KANSL1|42010|AP

STRADA|42962|RI

NASP|2754|ES

FAM86A|33885|ES

MX1|60665|AP

NPIPB5|94076|AD

C9orf3|86943|AT

ATXN2L|35848|ES

UGGT2|26130|AT

NADK|225|AP

ATP6V0B|2510|ES

RPL32|63469|AA

C6orf203|77126|AD

ZNF677|51701|AT

GTF2I|80087|ES

PTPMT1|15769|ES

DALRD3|64810|AA

NNT|71964|AP

OTUB1|16554|AP

DLST|28444|ES

INPP5F|13271|AP

SH3BP5|63561|AP

TBCEL|19169|AA

DYRK1B|49846|AA

SIAH1|36343|AT

SIAH1|36342|AT

PAX3|95775|AA

THAP6|69530|AT

SLC13A3|59695|AT

MARK2|16544|ES

MLH3|28471|ES

TANK|55736|AT

TANK|55733|AT

SERF1A|72415|AT

TIMM17B|89018|AA

PRKRIP1|81089|ES

MECR|1426|AA

PSME1|26851|RI

VWA8|25741|AT

VWA8|25742|AT

GAS8|38203|ES

C19orf24|46446|AP

C19orf24|46447|AP

FHL2|54829|ES

MTMR3|61690|ES

SCMH1|2057|ES

C11orf57|18727|RI

PQLC1|46266|ES

KIAA0895|79247|AT

GSTT1|61372|ES

FAU|16773|AD

NSUN5|97504|ES

ERCC1|50439|AP

PABPC1L|59497|ES

ZCCHC10|73327|AT

ZCCHC10|73326|AT

ZNF207|40205|ES

RNF43|42679|RI

SUOX|22340|ES

TMUB2|41807|ES

MIB2|181|AP

ZNF264|52205|AT

SEPT4|42703|ES

CPT1B|62869|AP

INPP4A|54627|AT

INPP4A|54628|AT

NECAB3|59001|RI

TMEM91|50048|RI

INTS10|82887|AD

HSPD1|56688|AP

ERCC1|50438|AP

ERBB3|22353|AP

RUNX2|76402|AT

MRPL52|26638|ES

FRG1|71414|ES

C9orf96|88041|AT

C9orf96|88040|AT

HDLBP|58346|AP

MRPL55|10086|ES

CSMD2|1687|AT

ALS2|56917|AT

ALS2|56916|AT

MARK3|29448|ES

ARHGEF1|50098|AP

DDX31|87987|AA

RBM19|24647|RI

CREBZF|18139|RI

NSUN4|2794|ES

PPIP5K2|72915|ES

COPS7A|19932|AP

COPS7A|19933|AP

GNAS|60005|ES

PPP1R3B|82592|AP

EFCAB5|40068|AT

RAB27A|30712|AP

RERE|543|AP

GLRX3|13473|AT

SAP130|55254|ES

PPP2R4|87853|ES

IDNK|86685|AP

ARL6IP4|25030|ES

ZSCAN32|33556|ES

SLC25A29|29249|AP

SPAG16|57329|AT

PSMB5|26691|ES

MBD1|45510|AA

SPATA9|72815|AT

CPNE1|59202|ES

SUGP2|48547|ES

TRAF3IP3|9682|AT

ARMCX6|89659|ES

UQCRQ|73320|ES

TAF6|80900|AD

PARP6|31525|ES

CACNB1|40626|AT

CACNB1|40627|AT

RCAN1|60488|AP

PLD3|49889|ES

SENP1|21406|AP

DIS3L2|57980|AT

HEXIM2|41951|AD

ATXN2L|35856|AA

C19orf82|47381|ES

ZNF544|52434|AD

SAR1B|73419|ES

NEK6|87524|AP

ZNF626|48724|AT

ZNF626|48723|AT

ELMOD3|54212|ES

RBP1|67033|AT

RPS25|19057|ES

PNKP|51102|AP

NLRP1|38721|AT

TRAPPC6A|50411|ES

ATHL1|13637|AP

TRRAP|80595|AA

POLM|79447|AA

PMEL|260567|ES

HKR1|49487|AA

GATSL3|61705|ES

EIF1AD|16975|ES

BNC2|85934|AT

BNC2|85935|AT

SLC25A29|29250|AP

TMEM205|47658|AA

RNF146|77449|ES

RANGRF|39163|RI

USP49|76147|AT

USP49|76148|AT

UBE3B|24315|AD

BCL7A|24921|AD

CCDC74A|55390|AA

CRYZL1|60450|AT

MED17|18329|RI

CECR5|60966|AP

ZNF519|44757|AT

ODF3B|62858|AA

WBP1L|12980|AP

WBP1L|12981|AP

EFCAB5|40069|AT

RHBDD1|57793|ES

MORN4|12731|AT

MORN4|12730|AT

MTRR|71541|AD

SLC25A1|61041|AP

SLC25A1|61042|AP

ANKRD42|18050|AT

NT5C3B|40959|AD

ZNF266|47344|RI

ARL6IP4|25029|AD

TCF3|46538|AA

SMUG1|22130|ES

FKBP1A|58490|AD

SLC30A5|72303|AA

TMTC2|23564|AT

CXorf40A|90313|AD

BCL11A|53659|AT

PTOV1|51095|AP

PTOV1|51094|AP

TELO2|33105|AA

TBC1D14|68729|AP

PI4KB|7599|ES

BRF1|29606|AP

EMC10|51201|ES

PRSS23|18201|AT

PRSS23|18202|AT

RABL2A|55066|AA

CWC25|40597|AD

SRSF11|3379|AA

NCOR1|39421|AA

PPP2R4|87831|AP

TUBB3|38175|ES

SEPSECS|68957|ES

U2AF1L4|49273|ES

MBD3|46524|AP

MBD3|46523|AP

TMTC2|23563|AT

ITGB3|42067|AT

GNB2L1|75072|ES

FILIP1L|65811|AT

SWI5|87728|AP

SWI5|87729|AP

TRPC4AP|59060|AA

KRTCAP3|53012|AP

HTATIP2|14707|AT

HTATIP2|14708|AT

NAP1L4|13939|ES

PUS3|19366|ES

DAB2IP|87446|RI

EPB41L1|59270|AA

RFX3|85744|AT

RFX3|85742|AT

SLC44A3|3818|AP

GABPB1|30575|AD

RARRES2|82227|RI

ZNF223|50269|AP

LAMA4|77299|RI

SCMH1|2056|ES

TBC1D10A|61715|AP

PMFBP1|37551|AT

TAMM41|63410|ES

SSR2|8163|ES

CIZ1|87711|AP

RPS9|51816|RI

DCLK1|25623|AT

SOX6|14503|ES

CORO1B|387275|ES

BZRAP1|42656|ES

TMEM25|19007|AT

TMEM25|19009|AT

IDNK|86684|AP

YIF1B|49603|AP

GPD2|55624|AP

MAP3K3|42943|AD

LIMCH1|69114|AT

LIMCH1|69113|AT

SUMF2|79803|ES

GUCD1|61406|AP

SULT1A1|35819|ES

TMUB2|41797|ES

FOXJ3|2067|AP

ATP8B2|7828|AP

KCNC4|4100|ES

ZMAT5|61664|ES

RXRA|88095|AP

RXRA|88094|AP

CAMTA2|38627|AP

HDAC9|78891|AT

RGS19|60190|AP

SLC44A3|3817|AP

MARS|22594|AT

MARS|22593|AT

ANKRD11|38081|ES

CYB561A3|16166|RI

RNASE1|26468|ES

TAF6|80901|AD

RPL34|70298|AT

RPL34|70300|AT

ABHD14B|65145|ES

ACIN1|26705|AD

MFSD12|46695|AT

MFSD12|46697|AT

AHCYL2|81742|AP

GTF2H2|72439|ES

RPS6KA3|88670|AP

CYFIP1|29679|AA

EEF1D|85446|ES

NPIPB5|35568|ES

NAP1L1|23480|ES

DNMT3A|52854|AP

GTPBP3|48288|AA

LRP2BP|71337|AP

BTBD3|58697|RI

USP19|64837|ES

BBIP1|13098|ES

STAU1|59740|ES

ZNF213|33457|AA

JMJD6|43621|ES

ERMARD|78488|AA

NUCB2|14519|AP

SNCA|69933|AD

CDKL3|73366|AT

ATP5G3|56093|RI

ARHGEF18|47101|AP

MFSD12|46699|ES

ACOX3|68766|AP

ZSCAN30|45154|AT

THEM4|7677|ES

MEF2D|8271|AP

MEF2D|8272|AP

KDM3A|54435|RI

ACBD4|41948|RI

SIAH1|36339|AP

POLM|79451|ES

KANSL1L|57261|AT

KANSL1L|57260|AT

ABCD4|28396|ES

UXS1|54857|AA

PPRC1|12938|ES

SH2B1|35871|AD

PRKRA|56158|AP

XYLB|64044|AT

XYLB|64043|AT

ELMOD3|54248|ES

PQBP1|89029|AD

MCCC1|67777|ES

NEDD4L|45660|ES

YIF1B|49604|AP

PIK3R2|48396|AT

PIK3R2|48398|AT

FBXW8|24672|AD

PRKCSH|47710|AD

C9orf9|87994|RI

MLPH|58113|ES

NSMF|88315|AA

GUCD1|61407|AP

FRG1|71415|ES

CALD1|81858|AP

ANAPC11|44207|ES

HDAC6|89007|AP

BRD9|71463|ES

SEMA4D|86804|AP

GLYR1|33863|AA

RBP1|67032|AT

KCNC3|51173|AT

NUP62|51126|ES

PTCD1|80624|AP

PTCD1|80625|AP

ANKRD54|62161|AP

FHL2|54834|ES

MBD1|45512|AA

FAHD2B|134675|ES

MBD1|45508|RI

SIPA1|16889|AP

QSER1|14865|AP

GTF2H2C|72400|AD

COX6C|84676|AT

C1S|20067|AP

SCLY|58199|AT

SCLY|58198|AT

SLC25A29|29253|RI

RBBP5|9524|AD

USP19|64840|AA

DIP2A|60939|AT

THTPA|26760|RI

COA1|79345|ES

NFE2L2|56128|AP

NECAB2|37804|ES

NUCB2|14520|AP

PNISR|77055|RI

ARSA|62899|RI

BRD9|71471|AA

KCNC3|51172|AT

TMX2|15923|AA

SYNPO|74101|AP

SUV420H1|17294|AT

SUV420H1|17293|AT

SNRPN|29703|AA

HAGH|33147|ES

RPS9|211189|ES

CSTF3|14884|AT

CDKN2A|86000|AP

NEIL2|82636|AD

XKR6|82615|AT

R3HCC1L|12758|ES

ACAT2|78314|AP

BMP1|82990|ES

CANT1|43973|RI

NKRF|89958|AP

HPS4|61509|ES

NDUFS7|46465|RI

DGUOK|54014|ES

PAPD4|72635|AA

ARFIP1|70858|ES

ATP5J|60268|ES

TPI1|19991|AD

ARHGEF7|26288|ES

GABPB1|30572|AT

TLE3|31418|AD

GABPB1|30573|AT

FBXL12|47415|AP

PRKCA|43100|AT

PRKCA|43101|AT

RAB7L1|9567|RI

SCMH1|2050|ES

CCM2|79585|ES

TTLL5|28526|ES

IST1|37509|AP

FAM63A|7537|AA

POU6F1|21806|AP

PNKP|51101|AP

CRCP|79874|ES

PPP2R4|87830|AP

KCTD6|65458|AP

HPS4|61510|AD

ZKSCAN5|80654|AP

ACAT2|78315|AP

ZNF44|47791|AT

NEDD4L|45674|ES

RGS19|60191|AP

KXD1|48456|AP

TCTN1|24462|AA

KLHL24|67806|ES

MIA|95220|AP

PRKACB|3585|AP

THAP2|23408|AT

THAP2|23409|AT

PRX|49900|RI

MARK2|16543|ES

MARK3|29447|ES

THRA|40841|AA

VRK2|53645|ES

C2orf43|52788|ES

SMEK1|28880|AD

CABYR|44864|AD

PCSK5|86632|AT

TMEM63B|76351|AP

FAM72A|9576|AP

FAM72A|9575|AP

RGS14|74759|ES

RRBP1|131582|ES

NUDT6|70521|AP

SON|60438|ES

TRIM5|14074|RI

CSTF3|14883|AT

VLDLR|85739|ES

PRUNE|7545|ES

NPIPA8|34240|ES

CREM|11239|AA

TRAPPC6A|50413|AD

POLL|12897|RI

TMEM150A|54305|ES

TIA1|53868|AT

MRPL55|10167|ES

SNRPA1|32758|ES

PQLC1|94856|ES

IPP|2771|AT

IPP|2772|AT

RALGAPA1|27239|ES

POU6F1|21805|AP

C14orf159|28847|AD

TVP23C|39355|AT

CALU|81712|ES

GSTO2|13052|ES

SIMC1|74640|ES

FOXP4|76113|AA

ZNF550|52294|ES

MADD|15713|AP

OCEL1|48242|AP

OCEL1|48243|AP

DCAKD|41928|AP

EP400NL|25239|AP

PRMT2|60964|ES

ALDH6A1|28366|AP

ALDH6A1|28367|AP

U2AF1L4|49270|ES

ADAM15|7904|ES

RNF111|30927|AA

FANCL|53651|AT

FANCL|53652|AT

ATF7|22092|AP

C17orf58|43118|AD

CASP7|13168|AP

ISY1|66700|ES

IST1|37514|AP

JAM2|60253|AT

JAM2|60254|AT

ZNF397|45144|AT

DCAF11|26842|AD

CTNND1|15998|ES

TRMT2B|89622|AT

TRMT2B|89623|AT

MRPL48|17729|ES

IL17RC|63262|ES

TMEM106C|21391|AD

MYO1C|38307|AP

TCEB1|84198|AP

TCEB1|84196|AP

KIAA1598|13241|AT

CLCC1|3983|ES

TCEB2|33302|RI

FBXO4|71890|ES

RSU1|10879|ES

MRPL21|17344|AD

B4GALT3|8588|AA

SPATA9|72817|AT

PLEKHM3|57205|AT

GLIPR1|23464|RI

HHLA3|3406|ES

ARMCX2|89666|AD

MAPK9|75012|ES

FPGS|87667|AP

FPGS|87668|AP

MAST4|72283|AT

BPTF|43116|AD

BATF2|16724|AP

SRRM1|1129|AD

ZHX1|85050|AP

PIGL|39428|AT

ADAMTSL4|7486|AT

ADAMTSL4|7487|AT

PUF60|85503|ES

PRR14|36165|AP

PRR14|36164|AP

ANKRD29|44841|AT
