# Supplementary material for: Immunological significance of survival-related alternative splicing in uveal melanoma
Source: Aging (Albany NY). 2022 Jan 19;14(2):811–25. doi: 10.18632/aging.203842 (PMC8833124; doi:10.18632/aging.203842)
Supplement: Supplementary Table 2 [file aging-14-203842-s002.doc]

**Supplementary Table 2. A total of 404 splicing factors (SFs).**

ID

ACIN1

AGGF1

ALYREF

AQR

ARGLU1

BAG2

BCAS1

BCAS2

BUB3

BUD13

BUD31

C17orf85

C19orf43

SDE2

C1QBP

C9orf78

CACTIN

CCAR1

CCDC12

CCDC130

CCDC75

CCDC94

CD2BP2

CDC40

CDC5L

CDK10

CDK11A

CDK12

CELF1

CELF2

CELF3

CELF4

CELF5

CELF6

CFAP20

CHERP

CIRBP

CLASRP

CLK1

CLK2

CLK3

CLK4

CLNS1A

CPSF6

CRNKL1

CSN3

CTNNBL1

CWC15

CWC22

CWC25

CWC27

CXorf56

DDX1

DDX17

DDX18

DDX19A

DDX19B

DDX20

DDX21

DDX23

DDX26B

DDX27

DDX39A

DDX39B

DDX3X

DDX3Y

DDX41

DDX42

DDX46

DDX5

DDX50

DDX6

DGCR14

DHX15

DHX16

DHX30

DHX34

DHX35

DHX36

DHX38

DHX40

DHX57

DHX8

DHX9

DNAJC6

DNAJC8

EEF1A1

EFTUD2

EIF2S2

EIF3A

EIF4A3

ELAVL1

ELAVL2

ELAVL3

ELAVL4

FAM32A

FAM50A

FAM50B

FAM58A

FMR1

FRA10AC1

FRG1

FUBP1

FUBP3

FUS

GEMIN2

GEMIN5

GNB2L1

GPATCH1

GPATCH3

GPATCH8

GPKOW

GRSF1

HNRNPA0

HNRNPA1

HNRNPA2B1

HNRNPA3

HNRNPAB

HNRNPC

HNRNPCL1

HNRNPD

HNRNPDL

HNRNPF

HNRNPH1

HNRNPH2

HNRNPH3

HNRNPK

HNRNPL

HNRNPLL

HNRNPM

HNRNPR

HNRNPU

HNRNPUL1

HNRNPUL2

HSPA1A

HSPA1B

HSPA5

HSPA8

HSPB1

HTATSF1

IGF2BP3

IK

ILF2

ILF3

INTS1

INTS3

INTS4

INTS5

INTS6

INTS7

ISY1

JUP

KHDRBS1

KHDRBS3

KHSRP

KIAA1429

KIAA1967

KIN

LENG1

LOC649330

LSM1

LSM10

LSM2

LSM3

LSM4

LSM5

LSM6

LSM7

LSM8

LSMD1

LUC7L

LUC7L2

LUC7L3

MAGOH

MATR3

MBNL1

MBNL2

MBNL3

MFAP1

MFSD11

MOV10

MSI1

MSI2

MYEF2

NCBP1

NCBP2

NELFE

NKAP

NONO

NOSIP

NOVA1

NOVA2

NRIP2

NSRP1

NUDT21

NUMA1

PABPC1

PAXBP1

PCBP1

PCBP2

PCBP3

PCBP4

PDCD7

PHF5A

PLRG1

PNN

PPIE

PPIG

PPIH

PPIL1

PPIL2

PPIL3

PPIL4

PPM1G

PPP1CA

PPP1R8

PPWD1

PQBP1

PRCC

PRMT5

PRPF18

PRPF19

PRPF3

PRPF31

PRPF38A

PRPF38B

PRPF39

PRPF4

PRPF40A

PRPF40B

PRPF4B

PRPF6

PRPF8

PSEN1

PSIP1

PTBP1

PTBP2

PTBP3

PUF60

QKI

RALY

RALYL

RAVER1

RAVER2

RBBP6

RBFOX2

RBM10

RBM14

RBM15

RBM15B

RBM17

RBM22

RBM23

RBM25

RBM26

RBM27

RBM3

RBM39

RBM4

RBM42

RBM45

RBM47

RBM4B

RBM5

RBM7

RBM8A

RBMS1

RBMX

RBMX2

RBMXL1

RBMXL2

RNF113A

RNF20

RNF213

RNF34

RNF40

RNPC3

RNPS1

RNU1-1

RNU2-1

RNU4-1

RNU5A-1

RNU6-1

SAP18

SAP30BP

SART1

SEC31B

SF1

SF3A1

SF3A2

SF3A3

SF3B1

SF3B2

SF3B3

SF3B4

SF3B5

SF3B6

SFPQ

SKIV2L2

SLU7

SMN1

SMNDC1

SMU1

SNIP1

SNRNP200

SNRNP25

SNRNP27

SNRNP35

SNRNP40

SNRNP48

SNRNP70

SNRPA

SNRPA1

SNRPB

SNRPB2

SNRPC

SNRPD1

SNRPD2

SNRPD3

SNRPE

SNRPF

SNRPG

SNRPN

SNU13

SNURF

SNW1

SPEN

SREK1

SRPK1

SRPK2

SRPK3

SRRM1

SRRM2

SRRT

SRSF1

SRSF10

SRSF11

SRSF12

SRSF2

SRSF3

SRSF4

SRSF5

SRSF6

SRSF7

SRSF8

SRSF9

SSB

SUGP1

SYF2

SYNCRIP

TAF15

TCERG1

TFIP11

THOC1

THOC2

THOC3

THOC5

THOC6

THOC7

THRAP3

TIA1

TIAL1

TNPO1

TOE1

TOP1MT

TOPORS

TRA2A

TRA2B

TRIM24

TTC14

TXNL4A

U2AF1

U2AF1L4

U2AF2

U2SURP

UBL5

USP39

WBP11

WBP4

WDR77

WDR83

WTAP

XAB2

YBX1

YBX3

ZC3H11A

ZC3H13

ZC3H18

ZC3H4

ZC3HAV1

ZCCHC10

ZCCHC8

ZCRB1

ZFR

ZMAT2

ZMAT5

ZMYM3

ZNF131

ZNF207

ZNF326

ZNF346

ZNF830

ZRSR1

ZRSR2
